# Solvent-Driven Self-Assembly of One-Dimensional Lepidocrocite Titanium-Oxide-Based Nanofilaments

**DOI:** 10.1021/acs.nanolett.4c00921

**Published:** 2024-05-22

**Authors:** Gregory R. Schwenk, Adam D. Walter, Michel W. Barsoum

**Affiliations:** Department of Materials Science and Engineering, Drexel University, 3141 Chestnut Street, Philadelphia, Pennsylvania 19104, United States

**Keywords:** Low-Dimensional, Self-Assembly, Morphology, Gelation, Lepidocrocite, Titanate

## Abstract

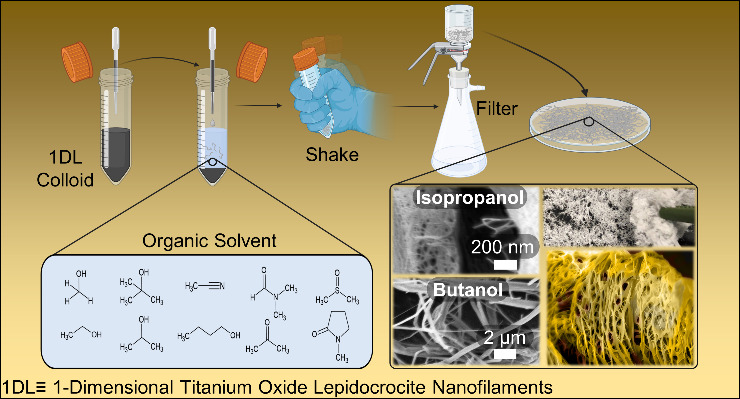

Herein, the self-assembly of one-dimensional titanium oxide lepidocrocite nanofilaments in 10 different water miscible organic solvents was investigated. The nanofilament snippets, with minimal cross sections of ∼5 × 7 Å^2^ and lengths around 30 nm, begin as an aqueous colloidal suspension. Upon addition, and brief mixing, of the colloidal suspension into a given solvent, a multitude of morphologies—seemingly based on the hydrophilicity and polarity of the solvent—emerge. These morphologies vary between sheets, highly networked webs, and discrete fibers, all with no apparent change in the lepidocrocite structure. On the micro- and nanoscale, the morphologies are reminiscent of biological, rather than inorganic, materials. The results of this work give insight into the self-assembly of these materials and offer new pathways for novel macrostructures/morphologies assembled from these highly adsorbent and catalytically active low-dimensional materials.

Lepidocrocite titanates (LTs) are layered structures that are comprised of edge-sharing TiO_6_ octahedra that require cations to exist between the layers for charge balance.^1,2^ LTs can undergo cation exchange, which expands their versatility over titania, their neutral counterpart, to include important applications like dye sensitized solar cells^3^ and batteries.^4^ By exchanging metal ions for the native cation from the synthesis, LTs can act as catalyst supports for the incoming ion.^5^ Like titania, they can also be used as photocatalysts.^6^ This, by no means, is an exhaustive list of applications.

Akin to this work, low-dimensional LT materials are available in a variety of morphologies like sheets,^7^ nanowires,^8,9^ nanotubes,^10−13^ and nanoribbons.^14^ However, though morphologies vary, most of their synthetic protocols borrowed from work by Kasuga and co-workers,^15,16^ require hydrothermal environments,^10,17^ organic additives,^9,18^ or expensive and unique precursors.^14^ Up to this point, controlling dimensionality—let alone morphology—has proven to be difficult.

Recently, a material similar to LTs, but truly one-dimensional^19^—growing tens of nanometers long with *subnanometer* cross sections—was discovered.^20,21^ A density functional theory (DFT) resolved structure (Figure 1A–C) was generated for this material that highlights its extreme dimensionality (Figure 1D). Owing to its unique phase and extreme dimensionality, these materials are henceforth referred to as one-dimensional lepidocrocite titanium oxide nanofilaments (1DLs). Notably, the experimental processing technique of 1DLs (Figure S1) is remarkably simple, employing nearly any^20^ Ti-based precursor (i.e., carbides, borides, nitrides, etc.), ambient pressure, temperatures of ∼80 °C, and common polyethylene reaction bottles.

**Figure 1:**
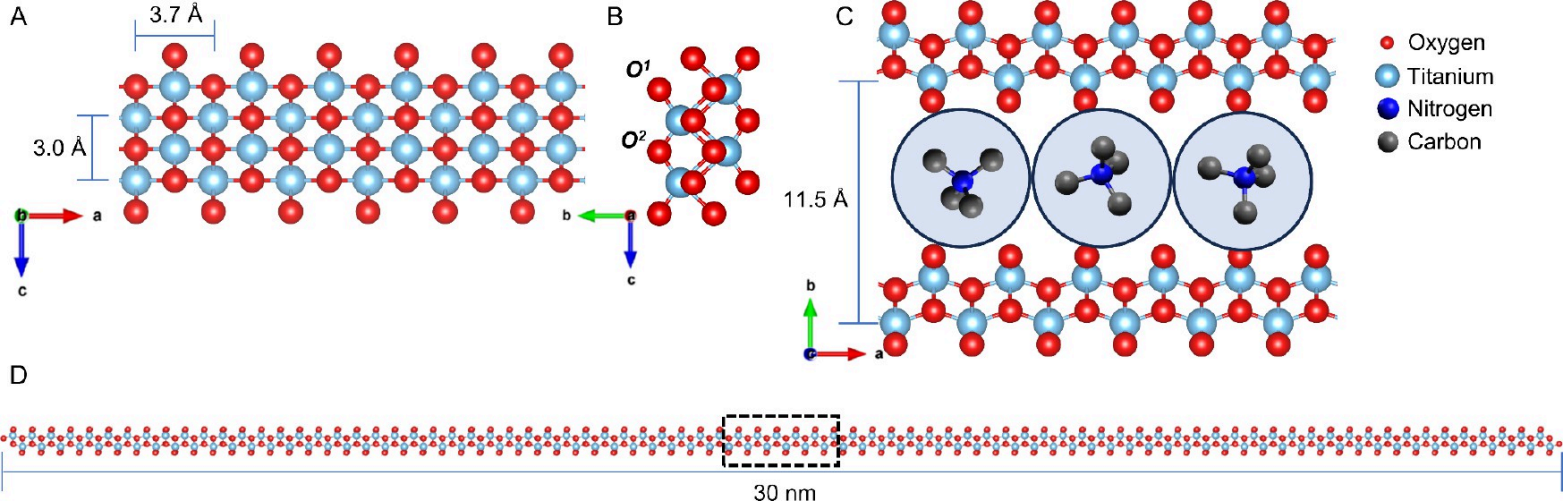
DFT resolved structure and lattice parameters of 1DLs. (A) *a*–*c* plane. (B) *b*–*c* plane, (C) *a*–*b* plane showing hydrated TMA^+^ between filaments that stack in the *b* direction. The circle around each TMA^+^ represents a hydration shell obtained from molecular dynamics simulations.^30^ (D) Typical 1DL strand showcasing the aspect ratio. The section shown in C is highlighted by dashed box in D. Note: A, B, and C panels are drawn to scale using van der Waals radii. H atoms are omitted for clarity.

Importantly, what distinguishes 1DLs from other LTs is their dimension in the *c* direction (Figure 1A) which is a result of their synthesis conditions. In the LT literature, the narrowest dimension reported in this direction is ∼10 nm^7^. In many other studies, it is much wider, which, in some cases, results in tubular morphologies.^10−13^ Notably, 1DLs require the presence of tetramethylammonium hydroxide (TMAOH) during formation, and when alkali hydroxides are used instead of TMAOH, phases similar to the alkali LTs reported in the literature^4,8^ are obtained.^22^ As such, it is believed that the TMA^+^ cation acts as a templating agent,^23^ upon which the formation of 1DLs depends.

Possessing similar properties to other LTs, but with dramatically enhanced surface areas from their extreme dimensionality, 1DLs have demonstrated efficient ion exchangeability among organic and inorganic cations alike and even sensitization effects by common textile dyes.^24−26^ Additionally, they have been investigated for use as sulfur hosts in lithium–sulfur batteries,^27^ among other applications.^20^ It is also notable that 1DLs may be assembled into various morphologies, like micron-scale 3D mesostructured particles^25,28^ and films comprised of mostly amorphous 2D sheets,^29^ without compromising on their underlying structure.

Since these materials are 1D, they are essentially *all* surface, presenting two primary types of O environments—both bridging (O^2^) and terminal (O^1^)—denoted in Figure 1B. In accordance with the literature,^2,4^ TMA^+^ cations are presumably localized between O^2^ sites on adjacent 1DLs. However, the potential for protons to localize on these terminations cannot be dismissed. Regardless, the high surface area with such hydrophilic constituents allows 1DLs to form remarkably stable colloidal suspensions in water with minimal effort, unlike their alkali LT analogs. This work demonstrates that these colloidal suspensions, which can be considered as TiO-based polymeric chains dispersed in water, can be manipulated to induce the formation of 1DL-based networks. By converting the colloidal media from pure water to various solvent systems, the 1DLs assemble in different ways that govern their overall final morphologies. In contrast to this simple assembly route, literature reports on the assembly of low-dimensional inorganic materials into networks often require the use of organic ligands anchored to the surface.^31^

Upon addition of the colloidal suspension into a variety of water miscible organic solvents, the 1DLs crash out and assemble into several macroscale morphologies. This occurs *near-instantaneously* upon shaking the mixture and forcing the interaction between the colloid and solvent (Supplemental Video S1). Much of the work discussed in this Letter achieved mixing by shaking the vessel by hand; however, for a more standardized protocol, a vortex mixer may instead be used (Supplemental Videos S2 and S3) while achieving the same final morphology (Figures S3 and S6). Since the assembly occurs so rapidly, it is believed that the resulting morphology is effectively governed by how well the solvent mixes with water. Essentially, this assessment of dispersion determines the size of the “nanobundles” the 1DLs assemble into and the nature in which they interact with each other—discussed in greater detail at the end of this Letter. The solids were formed by adding 2 mL of the colloidal 1DLs (concentration ca. 40 g/L) to 40 mL of the selected solvent. Each solvent was used as-received; they were chosen because of their general availability in academic laboratories, various functional groups, and varied degree of water miscibility.

Table 1 lists some properties of the solvents used, detailing the polarity indices, a relative comparison of hydrophilicity in the form of partition coefficients (log[*P*]), and their molecular structures. The solvent polarity indices, listed in column 2 of Table 1, are a composite measure of the solvent interactions with ethanol, dioxane, and nitromethane defined by Synder.^32^ The octanol/water log[*P*] value for each solvent^33^ is presented in column 3 of Table 1. This term serves effectively as a measure of a compound’s relative lipophilicity to hydrophilicity. The lower the value, the more hydrophilic.

**Table 1:**
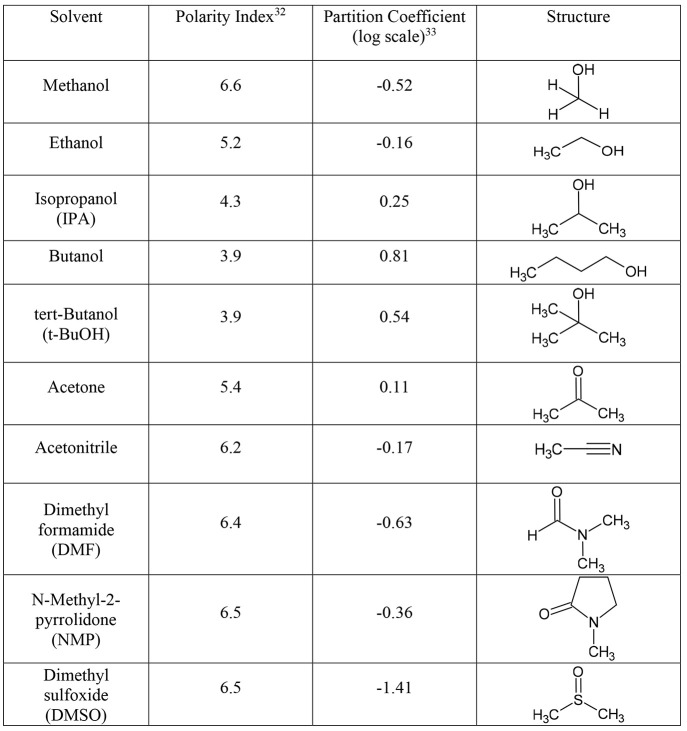
Summary of Solvents Used in This Study^a^

a Column 2: polarity index.^32^ Column 3: N-octanol/water partition coefficient on a log scale.^33^ Column 4: molecular structure.

In what follows, the macro, micro, and nano features of each 1DL colloid/solvent product are discussed—after they are dried via vacuum filtration—in the order the solvents are listed in Table 1. The variation and intricacies of the obtained morphologies are further highlighted in figures in the Supporting Information. Additionally, SEM micrographs of evaporated 1DL colloid, that was not exposed to any solvents, are shown in Figure S2.

Methanol produces a dense puck of flakes shown in Figure 2Ai, reminiscent of shed snakeskin. Figures 2Aii,iii and S3 show other SEM micrographs; from these, the 2D nature of the product is apparent. The lowest magnification micrograph in Figure S3 and nanoscale morphology (Figure 2Aiii) are reminiscent of crumpled 2D sheets and filtered films that are observed when colloidal suspensions of 1DL are vacuum dried,^20,21,29^ also shown in Figure S2.

**Figure 2:**
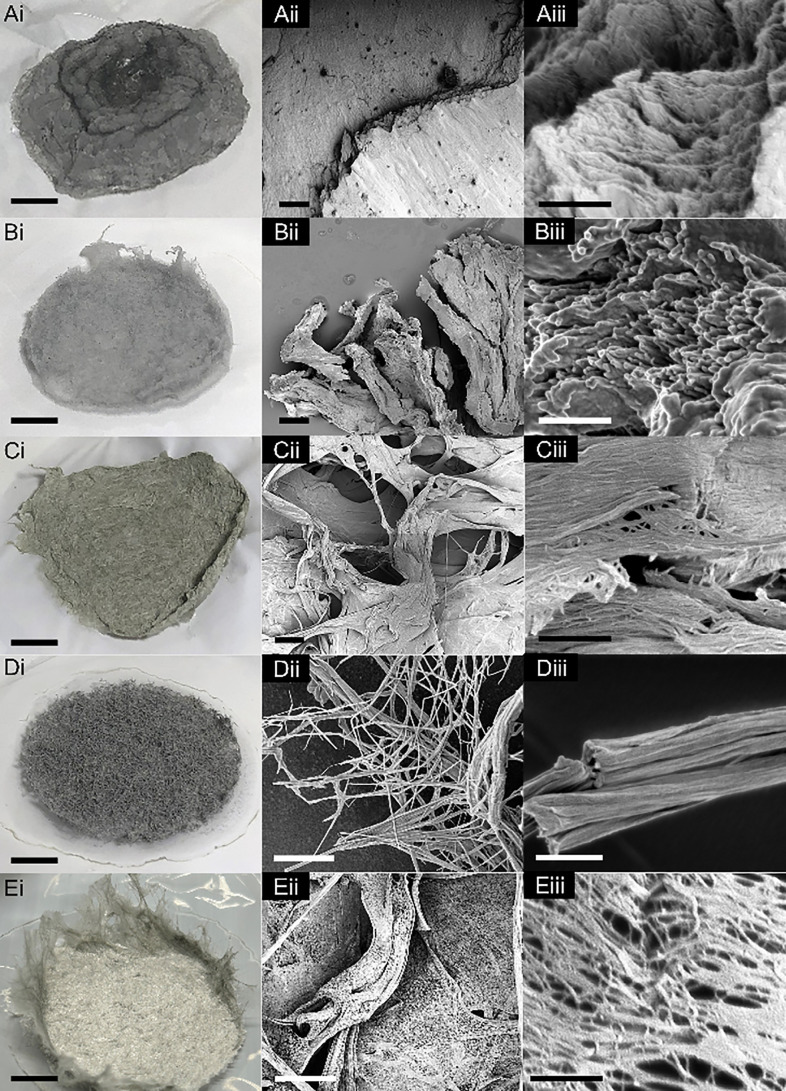
Photo- and micrographs of solids formed by adding 1DL colloids to a variety of alcohols. (A) methanol, (B) ethanol, (C) isopropanol (IPA), (D) butanol, and (E) tert-butanol (*t*-BuOH). Scale bars for column: i, 1 cm; ii, 10 μm; iii, 500 nm.

Ethanol produced a more fibrous amalgamation than methanol, but still dense (Figure 2Bi). This product exhibits an interesting morphology, that is nontrivial to describe in words, especially since the description is magnification-dependent. At low magnification (Figure 2Bii), there are no distinguishing features. However, at higher magnifications (Figures 2Biii and S4), bundles of nanofilaments appear, most easily visible at fracture surfaces.

Isopropanol (IPA) forms a fibrous product, similar to ethanol, but more voluminous; it also causes a slight yellowing in the solid (Figure 2Ci). At low magnification (Figure 2Cii), regions of both 2D sheets and fiber bundles appear. At increased magnification (Figure 2Ciii), micrometer-long bundles and large matted areas comprised of webbed nanofibers are clearly discernible. Figure S5 shows additional micrographs that further illustrate the microbundles and “nano-webs”.

Butanol produces thick fibers that appear like a brittle scouring pad (Figure 2Di). While IPA showed a combination of 2D and 1D morphologies, the overall impression one obtains from Figures 2Dii, iii, and S6 is solely based on 1D—i.e., fibers, braided ropes, and their intertwining.

The last alcohol tested, tert-butanol (*t*-BuOH), produced fine interwoven light gray structures that handled like paper (Figure 2Ei). Similar to IPA, *t*-BuOH results in a web-like nanostructure (Figure 2Eiii) that builds into microscale porous sheets (Figure 2Eii). Unlike IPA, however, these webs are not matted down, and the individual nanofilaments are visible throughout. As supported in Figure S7, the overall impression is one that showcases self-assembly of the fine snippets into quasi-2D structures and 1D bundles.

Acetone produced a dense consolidated mass of fibers that was brittle and flaky (Figure 3Ai). As shown in Figures 3Aii and S8, the morphology on the microscale includes quasi-2D sheets, lacey areas, and thick microbundles. Matted webbed networks of filaments also make up many regions of the solid. Some of these webs are spaced out enough to resemble nanoporous networks (Figure 3Aiii).

**Figure 3:**
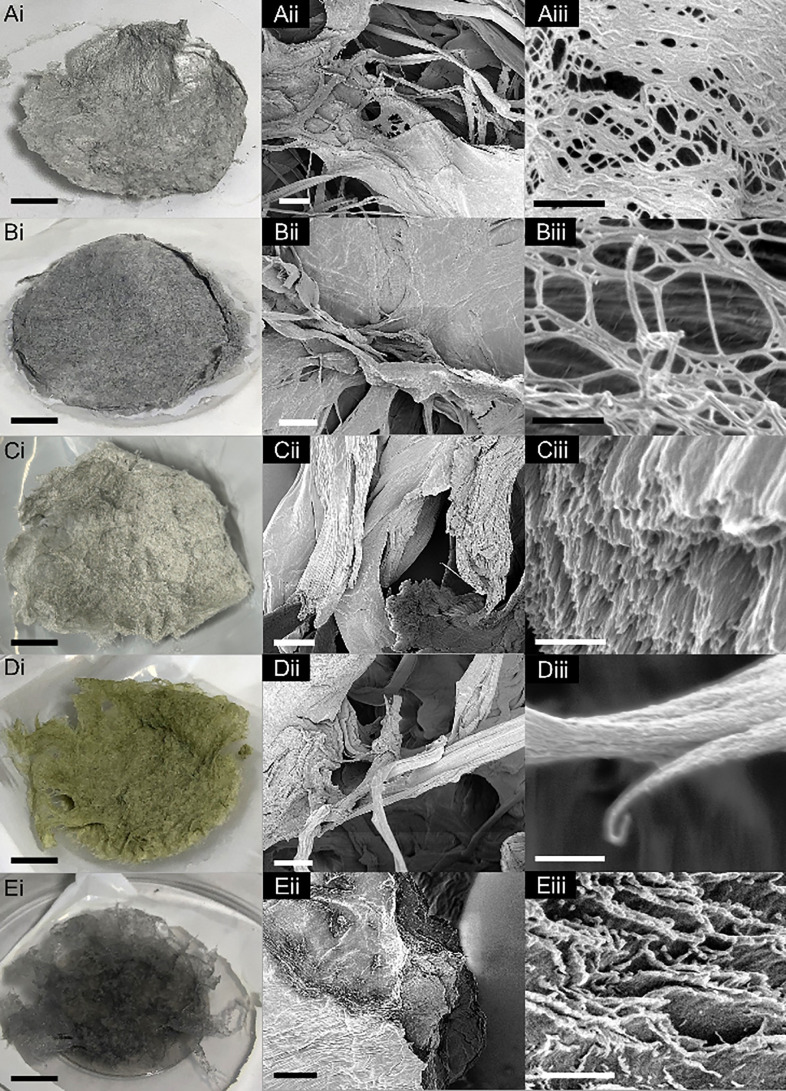
Photo- and micrographs of solids formed by adding 1DL colloids to a variety of nonalcohol solvents. (A) Acetone, (B) acetonitrile, (C) *N,N*-dimethylformamide (DMF), (D) *N*-methyl-2-pyrrolidone (NMP), and (E) dimethyl sulfoxide (DMSO). Scale bars for column i, 1 cm; ii,10 μm; iii, 500 nm.

Acetonitrile created a dried product (Figure 3Bi) with macro- and microscale morphologies that are similar to those of acetone (Figures 3Bii and S9). At low magnifications (Figure S9B), quasi-2D flakes comprised of microbundles are apparent. At higher magnifications, discrete snippets that make up the microporous sheets are visible (Figure 3Biii). These regions are primarily visible along edge regions and resemble stretched cheese cloth.

*N,N*-Dimethylformamide (DMF) formed a dense mass with little fibrous character (Figure 3Ci). DMF formed a sheeted product on the macroscale, which exhibits crumpled sheets on the microscale (Figures 3Cii and S10). Microbundles of 1D nanofilaments are shown at increased magnifications, especially at fracture surfaces (Figures 3Ciii), similar to the ethanol product.

*N*-Methyl-2-pyrrolidone (NMP) produced tightly packed fibers with a color change from gray to yellow-brown (Figure 3Di). The microscale morphology of the NMP product (Figures 3Dii and S11) is comprised in quasi-2D sheets and thick microbundles. These broad microfilaments appear to be comprised of a matted network of 1DL snippets braided together (Figure 3Diii).

Finally, dimethyl sulfoxide (DMSO) forms a film of flakes (Figure 3Ei). Since DMSO has a low vapor pressure, this film is difficult to dry fully. If dried at room temperature, some DMSO remains in the film, enabling flexibility. Supplemental Video S4 shows the film being bent and recovering to its original shape. This is in stark contrast to the extremely brittle films formed by drying the colloidal 1DL directly, that break easily when folded. On the microscale (Figure 3Eii and Figure S12), the morphology resembles the methanol product (Figure 2Aii). However, at higher magnification, the bundled filaments protrude out of the fracture surface (Figure 3Eiii).

A fascinating result of this work is the formation of a gel monolith (Figure S13) by adding concentrated (≈ 40 g/L) 1DL colloid to methanol in a ∼1:10 volume ratio, respectively. After adding the colloid to the methanol, the viscosity increases and fibers appear, faintly visible to the naked eye. Within a few minutes to hours—depending on the colloidal concentration—the monolith starts to take shape and by ∼24 h (depending on initial colloidal concentration), it separates from the container and forms a free-standing gel.

Once removed from the container, the monolith holds its shape (Figure S13) and is quite similar to hybrid inorganic gels—in the sense that it can restore itself from compression in the direction normal to its base, but it is brittle in any other direction. Additionally, when submerged in water, the gel floats until it loses shape over time and begins to break apart, resuspending the colloidal 1DLs. Notably, however, if the gel has been fully dried prior to being submerged, it will remain in the form of flakes and will not resuspend. With acetonitrile, a small amount of gel protruded from the resulting filaments (Figure S14) several weeks after initial fiber formation.

The formation mechanism of these monolithic gels is presently unknown and is the subject of ongoing work. Since the functionality of the nitrile group in acetonitrile varies significantly from the alcohol in methanol, it appears that gel formation is based on other solvent properties, such as polarity index or hydrophilicity. Along this line of thought, it is reasonably suspected that gelation may be induced from most of the tested solvents, not only methanol, so long as an optimal ratio of colloid to solvent—and thus appropriate polarity index and partition coefficient—is achieved. Such implications are currently being investigated as well as the mechanical, adsorptive, and photocatalytic properties of these gels.

Since the morphology of these assemblies varies greatly, several characterization techniques were employed to determine if the underlying structure remains consistent with 1DL. First, the Raman spectra of each of the films (Figure S15) show that the characteristic peaks of orthorhombic lepidocrocite^11^ (190 cm^–1^, 280 cm^–1^, 380 cm^–1^, 445 cm^–1^, 660 cm^–1^, 700 cm^–1^, 950 cm^–1^) remain relatively unchanged when compared to untreated 1DLs.^21^ Additionally, the X-ray diffraction (XRD) patterns (Figure S16) of each film show that the core structural peaks at 2θ = 48° and 62°, corresponding to the 200 and 002 planes, are present in each pattern.^24^ There are some peaks arising in the regions between ∼25° and 40° which are noncrystallographic in origin and correspond to the periodicity of interfilament orientations (Figure S17). Interestingly, the peak at ∼8°, corresponding to 020, varies only slightly between samples. This indicates that TMA^+^ cations are still present in between the 1DLs along the *b* direction, while the small variations in spacings are most likely a result of cation solvation effects.

The FTIR spectra of each film (Figures 4 and S18) confirm the remaining presence of TMA^+^, with the peaks at 951 cm^–1^, 1482 cm^–1^, and 3026 cm^–1^.^34^ There are few differences between the spectra resulting from alcohol treatments—indicating that, during drying, the alcohol was fully evaporated. Acetone and acetonitrile remain consistent with this observation as well. The less volatile solvents, DMF, DMSO, and NMP, show some peaks consistent with residual solvent.^35−37^ The DMF spectrum shows a peak at ∼1600 cm^–1^ that is absent in pure DMF but consistent with near-ambient dissociative adsorption of DMF onto a Ti^4+^ site in titania.^35^ The major takeaway of this work primarily lies in the morphologies achievable by simply changing the solvent system of 1DLs. It is apparent from the FTIR results (Figures 4 and S18) that the high vapor pressure solvents are eliminated during drying—indicating that the films, at that point, are comprised entirely of a network of 1DLs.

**Figure 4:**
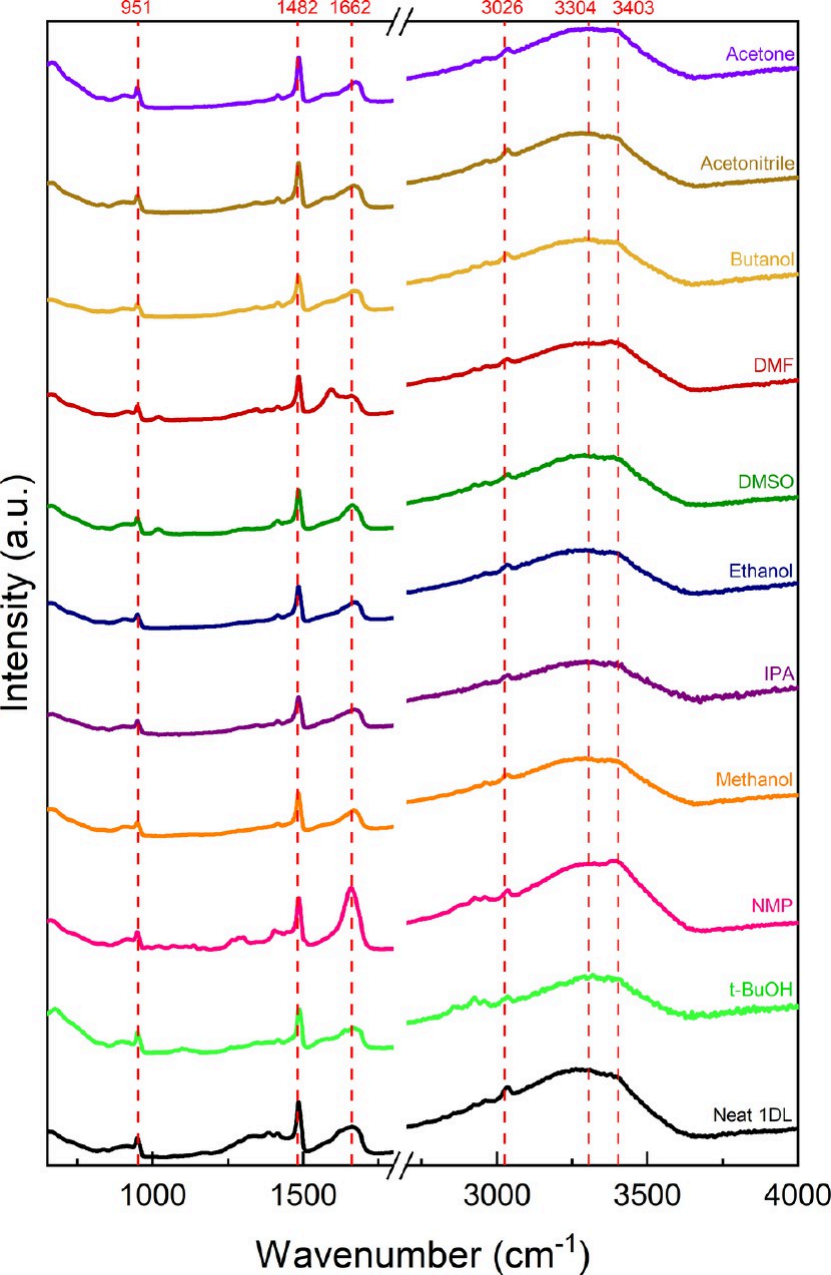
FTIR spectra of the various films produced in this study. Note the labeled peaks are unchanged throughout the samples—corresponding to either 1DLs or the intercalated TMA^+^. Zoomed-in regions of wavenumbers below the break are shown in Figure S18.

Looking through the lens of the water miscibility of each solvent, in terms of both polarity and log[*P*], a trend is evident. The more polar and lower log[*P*] solvents such as methanol (Figures 2Aii and S3), ethanol (Figures 2Bii and S4), DMF (Figures 3Cii and S10), and DMSO (Figures 3Eii and S12)—which would be efficient in removing water from between the 1DL snippets—form sheets on the microscale. The less polar and mid log[*P*] solvents, with reduced solvent exchange efficiency, like IPA (Figures 2Cii and S5), *t*-BuOH (Figures 2Eii and S7), acetone (Figures 3Aii and S8), and acetonitrile (Figures 3Bii and S9), form webbed structures. When the solvent molecules (like butanol and NMP) get bulky, they produce more spaced-out regions of bundles (Figures 2Dii, S6, 3Dii, and S11) seemingly independent of polarity and log[*P*]. As expected, nonpolar and water-immiscible, high log[*P*], solvents such as toluene and hexanes cannot induce any assembly, as the mixtures are biphasic, similar to oil and water.

To sum up the results at this juncture, the leitmotif that emerges from all the morphologies shown is the clear and unambiguous 1D nature of the product. This supports that the fundamental building blocks of all morphologies are the nanosnippets described above and shown schematically in Figure 1D.

It is likely that the change in solvent system directly influences the local H-bonding environment, which possibly accounts for the self-assembly. A schematic of the proposed formation mechanism is shown in Figure 5. This change in H-bonding environment leads to the near-instantaneous crashing-out of assemblies from the aqueous suspension of colloidal 1DLs (Figure 5A). For this to occur, the solvent must be miscible with water—to some degree—to allow for it to enter the space between adjacent filaments or bundles and displace some of the water (Figure 5B). If the 1DLs stay relatively hydrated, H-bonding is responsible for the 1D bundling of snippets (Figure 5C). However, if the water is removed completely, a dehydration reaction, resulting in oxolation and/or olation, could occur between adjacent filaments (Figure 5C).

**Figure 5:**
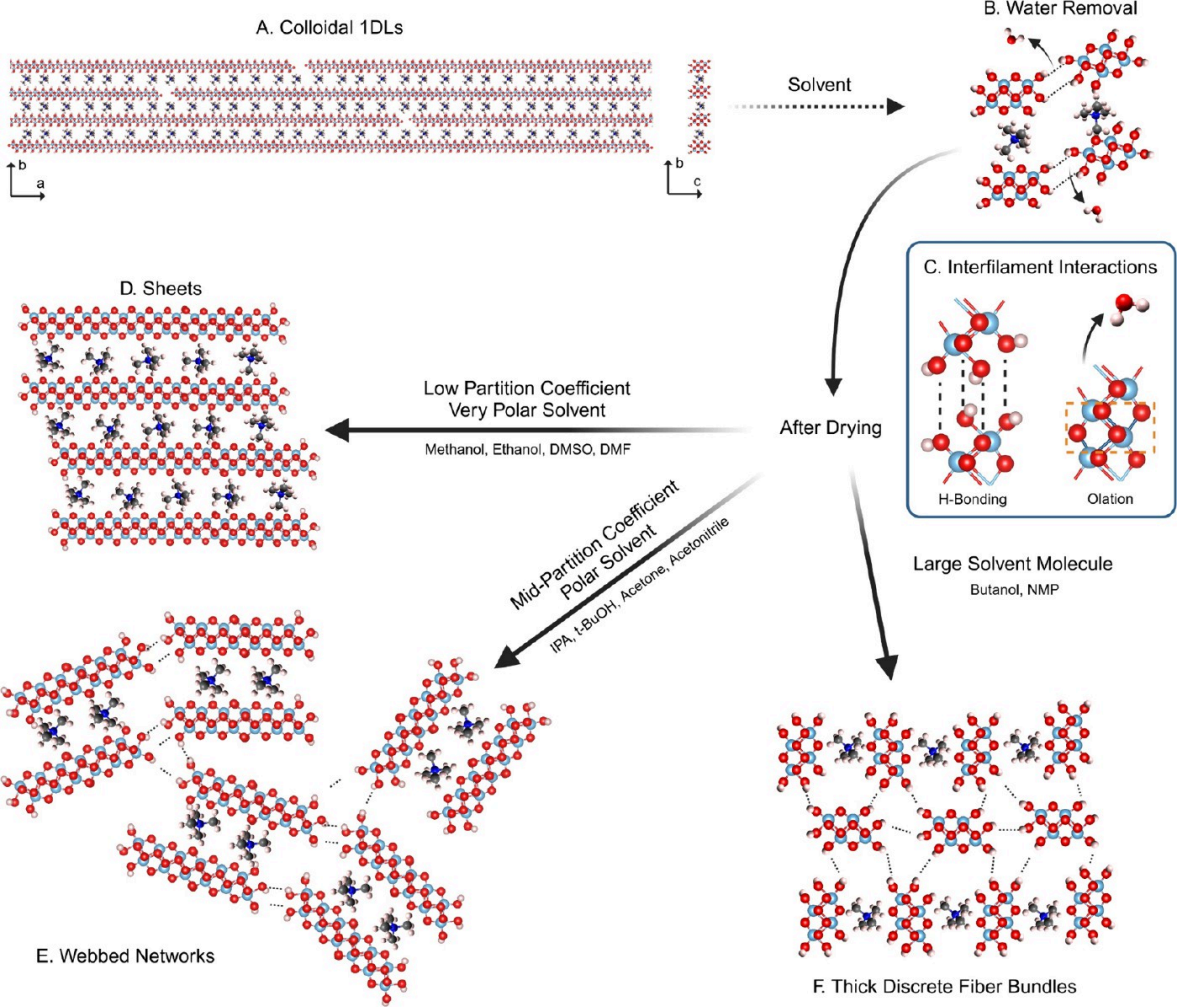
Proposed mechanistic explanation for the formation of the various morphologies encountered in this study. (A) Colloidal 1DLs are added to water miscible solvent. (B) Water is removed, and there is a local increase in (C) interfilament interactions. Based on the solvent, the relative amounts of H-bonding and olation change. Low log[*P*] and very polar solvents produce (D) sheets, primarily through olation. Mid log[*P*], but still polar, solvents produce (E) webbed networks, through a mixture of olation and H-bonding. Large solvent molecules remove the least amount of water, producing (F) thick discrete fibers, through mostly hydrogen bonding. Examples of each solvent are included next to its corresponding connecting arrow.

The more polar and hydrophilic the solvent molecule is, the more likely it is to exchange with water, leading to the near-instantaneous formation of bonds between adjacent snippets, producing sheets (Figure 5D). The same occurs when filtering and drying the aqueous 1DL colloid, from which dense 2D sheets are formed. The olation and/or oxolation must occur along the *c* direction, since TMA^+^ remains in the material, as shown by the characteristic TMA^+^ IR signatures that still exist after drying (Figure 4). As the solvent molecules get less polar and more lipophilic, they will have difficulty displacing water between *every* filament, but should still displace some of the water during drying, thereby forming smaller sheets–which coordinate via H-bonding producing webbed networks (Figure 5E). The larger molecules, regardless of polarity or hydrophilicity, experience difficulty completely surrounding the TMA^+^ cations, thus behaving like a pseudosurfactant forcing the more hydrophilic 1DLs into thick bundles (Figure 5F).

According to this conjecture, smaller molecules would allow for thinner bundles and, when more similar to water, sheets. In an effort to prove this behavior, 1DL was added to a mixture of equal parts of butanol and IPA. This resulted in one of the more fascinating microscale morphologies, completely different than the products from each solvent independently—resembling a highly networked mixture of discrete nanosized fibers among larger fibers. These micrographs are shown in Figure S19, and a photograph is shown in the TOC graphic. Further investigation into mixed solvents is warranted, but outside the scope of this Letter.

This work unequivocally demonstrates the extreme 1D nature of these materials and the resulting ability to produce a plethora of morphologies by simply changing the nature of the solvent into which a colloidal suspension (Figure S20) is injected. These results also shed light on the challenging subject of colloidal particle interactions, providing new entry points for theoreticians to test their models.^38^

Practically, the importance of this work cannot be overstated. In many applications where nanomaterials are considered, from adsorption to catalysis, among others, their morphology can be of the utmost importance. The ease by which 1DL can go from 2D sheets to fine-subnanometer meshes is truly remarkable and provides a new parameter to manipulate for inorganic materials and their relation between morphology and application. Producing these materials, at near ambient conditions, from ubiquitous, abundant precursors—using nothing more sophisticated that plastic bottles and a shaking incubator—is arguably paradigm shifting.

## Supporting Information Available

Mixing of aqueous colloidal 1DL with butanol by shaking (MP4)
Mixing of aqueous colloidal 1DL with methanol by vortex (MP4)
Mixing of aqueous colloidal 1DL with butanol by vortex (MP4)
DMSO/1DL film being manipulated by hand (MP4)
Detailed experimental methods, Raman spectra, X-ray diffraction patterns, and additional SEM images of all products (PDF)

## Abbreviations

LT: lepidocrocite titanate
1DL: one-dimensional titanium oxide lepidocrocite nanofilament
TMAOH: tetramethylammonium hydroxide
DFT: density functional theory
IPA: isopropanol
*t*-BuOH: tert-butanol
DMF: *N,N*-dimethylformamide
NMP: *N*-methyl-2-pyrrolidone
DMSO: dimethyl sulfoxide
