# Supplementary material for: Solvent-Driven Self-Assembly of One-Dimensional Lepidocrocite Titanium-Oxide-Based Nanofilaments
Source: Nano Lett. 2024 May 22;24(25):7584–92. doi: 10.1021/acs.nanolett.4c00921 (PMC11212056; doi:10.1021/acs.nanolett.4c00921)
Supplement: Supplementary file 5 — nl4c00921_si_005.pdf [file nl4c00921_si_005.pdf]

Supporting Information for: Solvent-Driven Self-Assembly of One-Dimensional Lepidocrocite Titanium Oxide Nanofilaments

Gregory R. Schwenk*,‡, Adam D. Walter‡ and Michel W. Barsoum*

Department of Materials Science & Engineering, Drexel University, 3141 Chestnut Street, Philadelphia, PA, USA, 19104
*Corresponding Authors (grs72@drexel.edu, barsoumw@drexel.edu), ‡These authors contributed equally

TABLE OF SUPPORTING FIGURES

Figure S1. Fabrication process of 1DL colloid. 3
Figure S2. SEM micrographs of 40 g/L 1DL colloid after vacuum filtration. 3
Figure S3. SEM micrographs of methanol product after vacuum filtration. (A), (C), and (E) were formed by mixing 1DL colloid and methanol by hand shaking. (B), (D), and (F) were formed by mixing 1DL colloid and methanol by vortex mixer. Note the similarities between the products formed by the different processing methods. 4
Figure S4. SEM micrographs of ethanol product after vacuum filtration. 5
Figure S5. SEM micrographs of isopropanol product after vacuum filtration. 6
Figure S6. SEM micrographs of butanol product after vacuum filtration. (A), (C), and (E) were formed by mixing 1DL colloid and butanol by hand shaking. (B), (D), and (F) were formed by mixing 1DL colloid and butanol by vortex mixer. Note the similarities between the products formed by the different processing methods. 7
Figure S7. SEM micrographs of tert-butanol product after vacuum filtration. 8
Figure S8. SEM micrographs of acetone product after vacuum filtration. 9
Figure S9. SEM micrographs of acetonitrile product after vacuum filtration. 10
Figure S10. SEM micrographs of dimethylformamide (DMF) product after vacuum filtration. 11
Figure S11. SEM micrographs of n-methyl-2-pyrrolidone (NMP) product after vacuum filtration. 12
Figure S12. SEM micrographs of dimethyl sulfoxide (DMSO) product after vacuum filtration. 13
Figure S13. Gel monolith produced by combining colloidal 1DL with methanol in a 1 to 4 volume ratio, respectively. This gel was produced in a 250 mL polyethylene bottle and removed for imaging. Diameter of gel is ≈5 cm. 13
Figure S14. Gel produced by combining colloidal 1DL with acetonitrile in a 1 to 4 volume ratio, respectively. Gel was produced in a 20 mL glass scintillation vial. The red oval surrounds the soft, self-standing portion of the sample. Solid below that monolith is quite soft and resembles tentacles. 14
Figure S15. Raman spectra of the various films produced in this study. Note the labeled peaks are relatively unchanged across the samples. Labeled peaks are standard issue lepidocrocite[3]. 15
Figure S16. XRD patterns of the various films produced in this study. Note the labeled peaks are relatively unchanged across the samples. Labels at the top indicate the indexed peak values. 16
Figure S17. Zoomed in mid-region of XRD shown in **Figure S17**. Note shifting peaks in this region signifying changes in the non-stacking order peaks. 17
Figure S18. Low wavenumber region of the FTIR shown in **Figure 4**. 18
Figure S19. SEM micrographs of 1/1 w/w isopropanol/butanol product after vacuum filtration. Both scale bars are 2 μm. 19
Figure S20. Photo of the concentrated (≈40 g/L) aqueous colloidal suspension of 1DLs. The suspension is a dark grey, bordering on black, with a high viscosity. 20

EXPERIMENTAL METHODS

1DL Colloid Fabrication (Diagram shown in Figure S1)

The 1DLs were fabricated by adding 10 g of titanium diboride (as-received 99.9%, - 325 mesh, Thermo Fisher Scientific Inc., Waltham, MA, USA) to 87.5 g of tetramethylammonium hydroxide (as-received 25% w/w aqueous 99.9999%, Alfa Aesar, Ward Hill, MA, USA) in a 250 mL HDPE bottle vented with a single 23-gauge needle. The bottle was heated and shaken in an incubator (Labnet International Shaking Incubator, NJ, US) at 200 rpm and 80°C for 4 days. The product sediment was combined with ethanol (as-received, 200 Proof, Decon Laboratories Inc., King of Prussia, PA, USA), vortex shaken, and centrifuged at 3,500 rpm for 2 min. The clear supernatant was discarded after each wash. This was repeated until a pH ~ 7 was achieved (usually 3 times). Ultrapure water (<18.2 mΩ/cm) was added to the ethanol washed product and the material was suspended by vortex mixing. The mixture was centrifuged at 5,000 rpm resulting in a highly stable colloidal suspension (**Figure S20**) while unreacted powders settled to the bottom. The colloidal 1DLs were used at ≈40 g/L. This concentration confirmed by vacuum filtering 2 mL of colloidal 1DL through a 25 μm thick microporous monolayer polypropylene membrane (Celgard® 3501, Celgard, NC, US) over a fritted glass filter apparatus. Once filtered, the solid was fully dried in an oven, under vacuum, at 80°C and the weight of the residue was measured.

Solid Formation

Films were formed by adding 2 mL of the concentrated (≈40 g/L) 1DL colloid to ≈40 mL of each solvent, or in some similar composition. The mixture was hand shaken, or vortex mixed, and vacuum filtered over a fritted glass filter apparatus to dry. The films were then allowed to dry fully in a fume hood for at least overnight before characterizing.

Material Characterization

The sample morphologies were imaged using a field emission scanning electron microscope, SEM (Supra 50VP, Carl Zeiss AG, Jena, DE). Due to the non-conducting nature of our samples, they were sputtered for 30 s at 40 mV with a Pt/Pd sputter-coater (208HR, Cressington Scientific Instruments, Watford, UK). Micrograph contrast adjustments and de-speckling were done ImageJ.

FTIR measurements were carried out with an IR spectrometer (PerkinElmer Spectrum ONE FT-IR with Universal UATR Sampling Accessory, Shelton, USA). The solid was loaded onto the attenuated total reflectance (ATR) crystal and ~50 force units were applied. IR activity was monitored in 650–4000 cm^{-1} range, after which the data was processed by an ATR correction provided by the instrument's software.

To acquire Raman spectra of the samples a small section of the film was loaded onto a glass microscope slide. A RM-2000 Vis Raman spectrometer (Renishaw, Wotton-under-Edge, England, UK) was used to probe the observed Raman shifts in each sample using a 633 nm beam source. The samples were exposed to a 100% laser power from wavenumbers of 150-1000 cm^{-1}. Due to an incited phase change as the result of previous work[1, 2], the spectra were acquired at 25% laser power before and after the 100% scan to confirm no phase change occurred. This was indeed the case.

X-Ray diffraction, XRD, patterns were acquired with a MiniFlex 600 benchtop XRD (Rigaku, Tokyo, Japan) equipped with a Cu-K_α radiation source. The films shown in **Figure 2** and **Figure 3** were taped onto the sample stage. Samples were scanned from 3 to 65° 2θ with step increments of $0.02 \circ s^{-1}$ and exposure times of 1.2 s/step.

.

SUPPORTING FIGURES

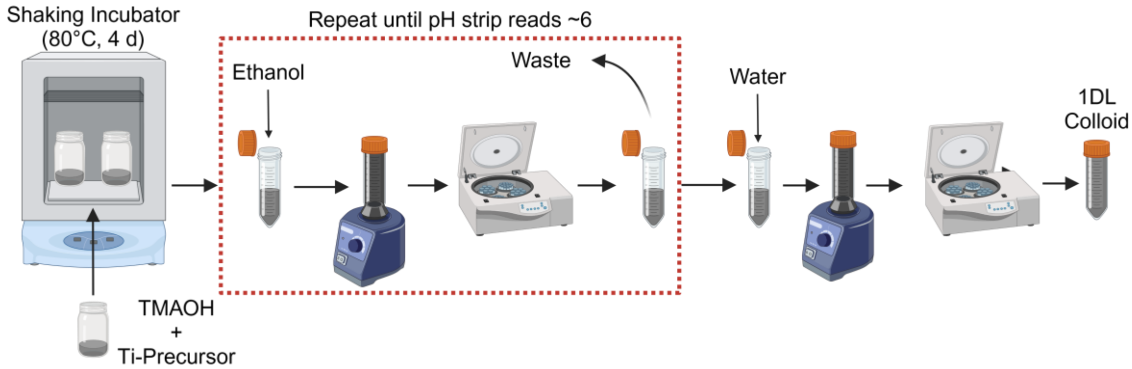

Figure S1. Fabrication process of 1DL colloid.

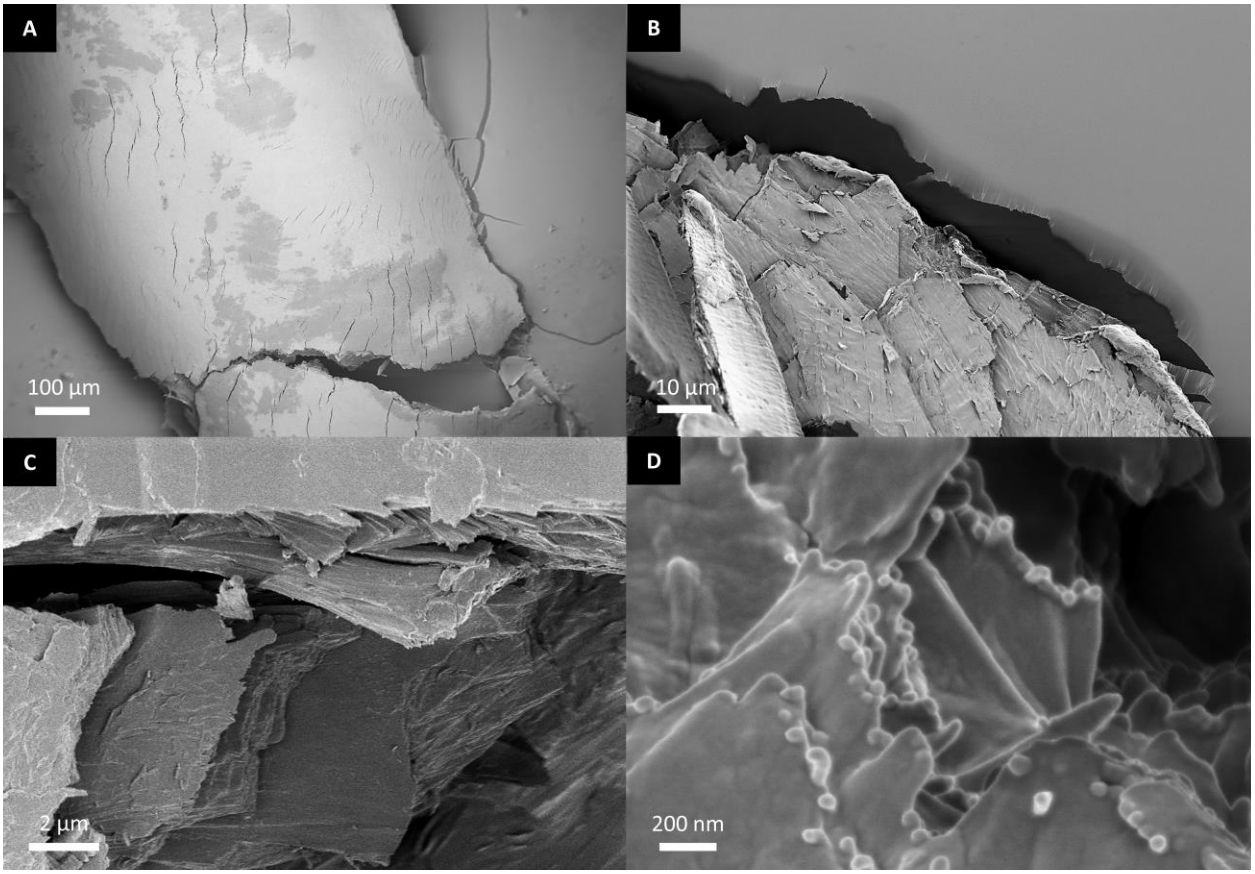

Figure S2. SEM micrographs of 40 g/L 1DL colloid after vacuum filtration.

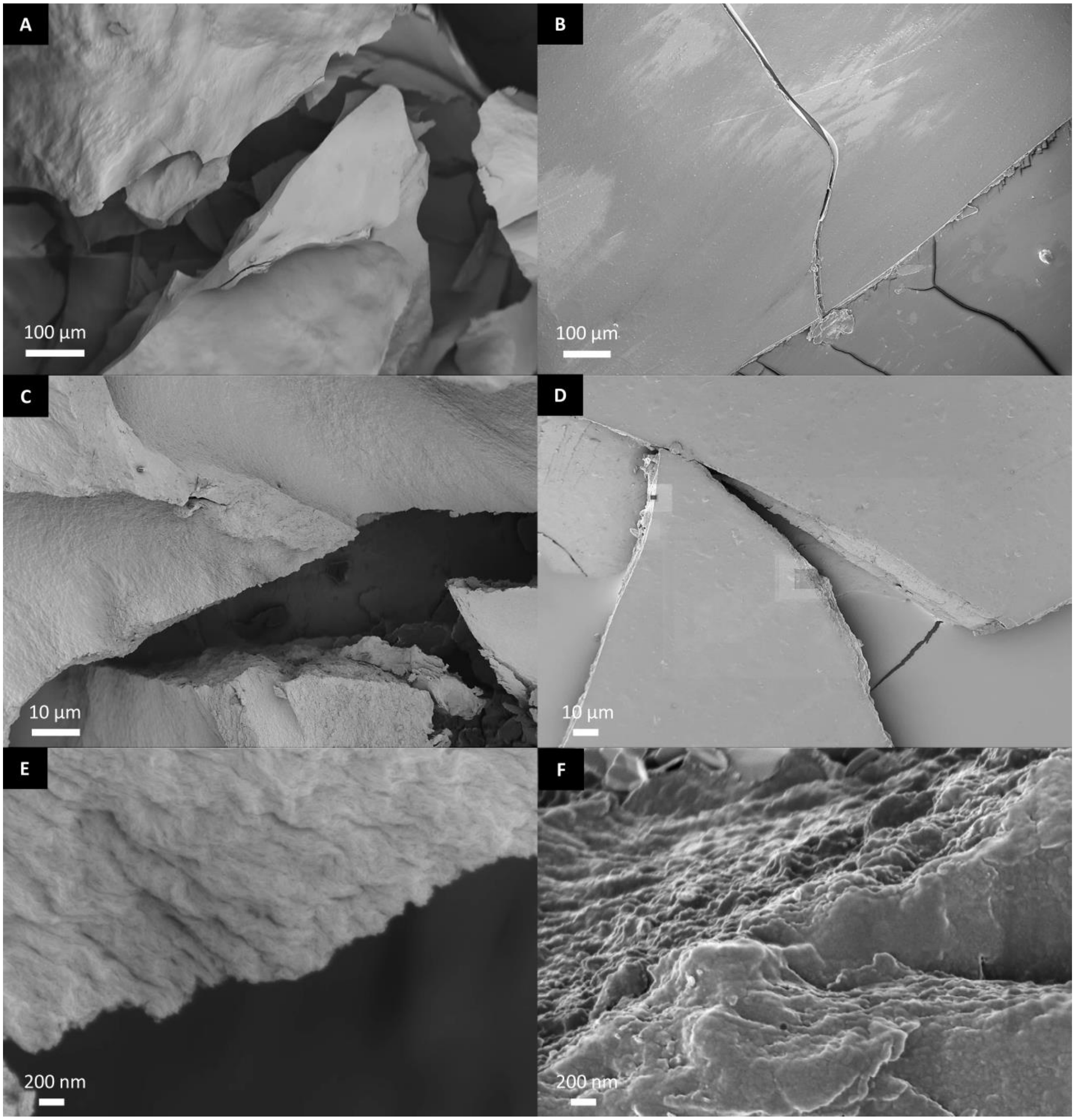

Figure S3. SEM micrographs of methanol product after vacuum filtration. (A), (C), and (E) were formed by mixing 1DL colloid and methanol by hand shaking. (B), (D), and (F) were formed by mixing 1DL colloid and methanol by vortex mixer. Note the similarities between the products formed by the different processing methods.

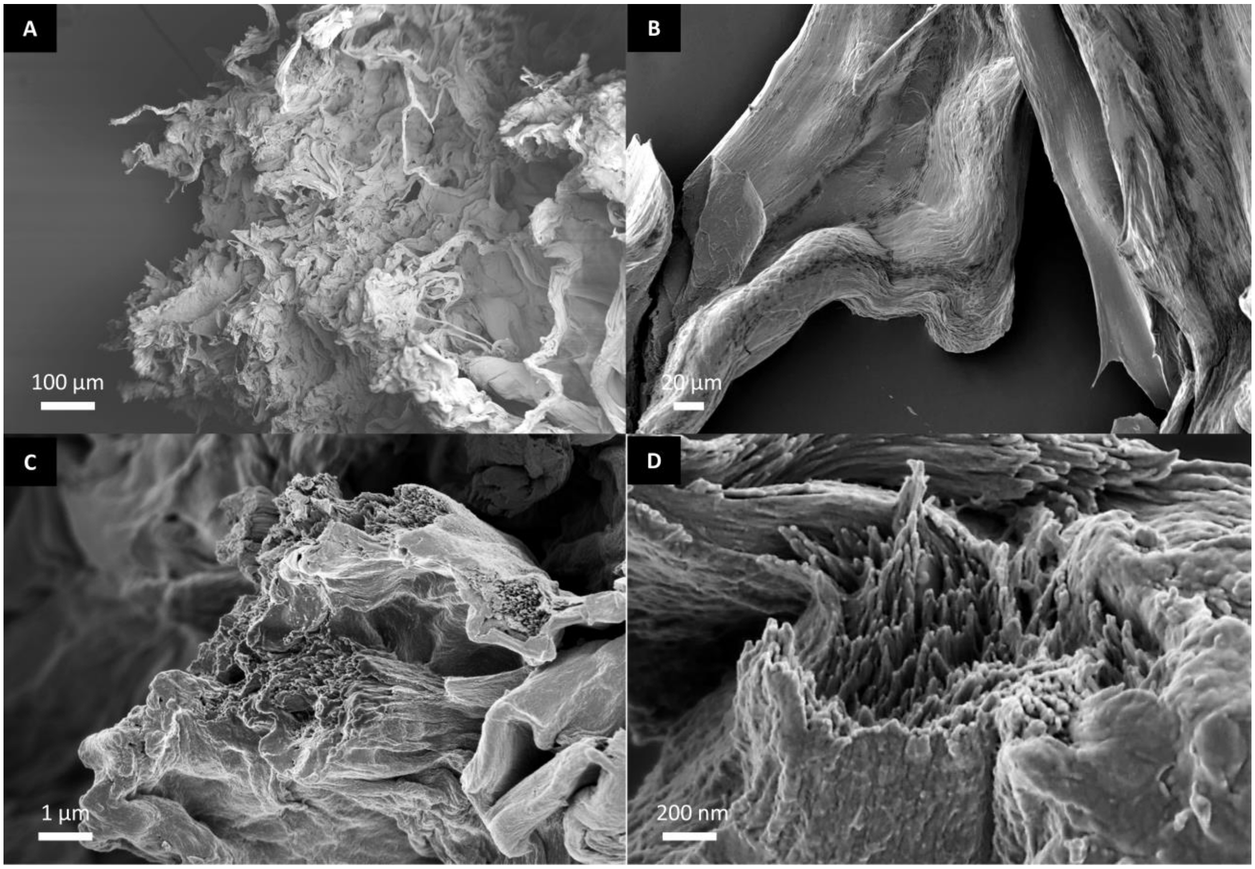

Figure S4. SEM micrographs of ethanol product after vacuum filtration.

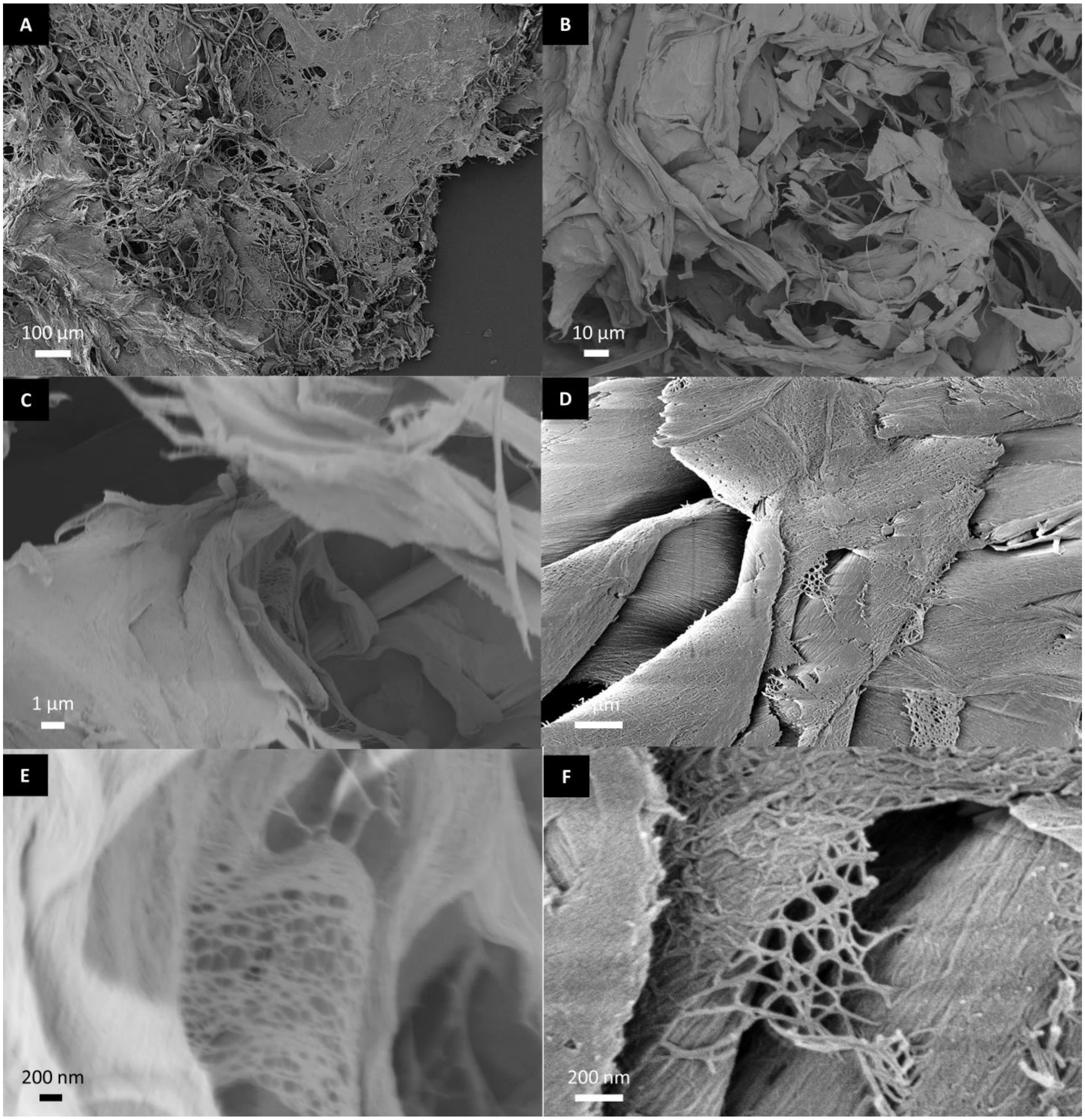

Figure S5. SEM micrographs of isopropanol product after vacuum filtration.

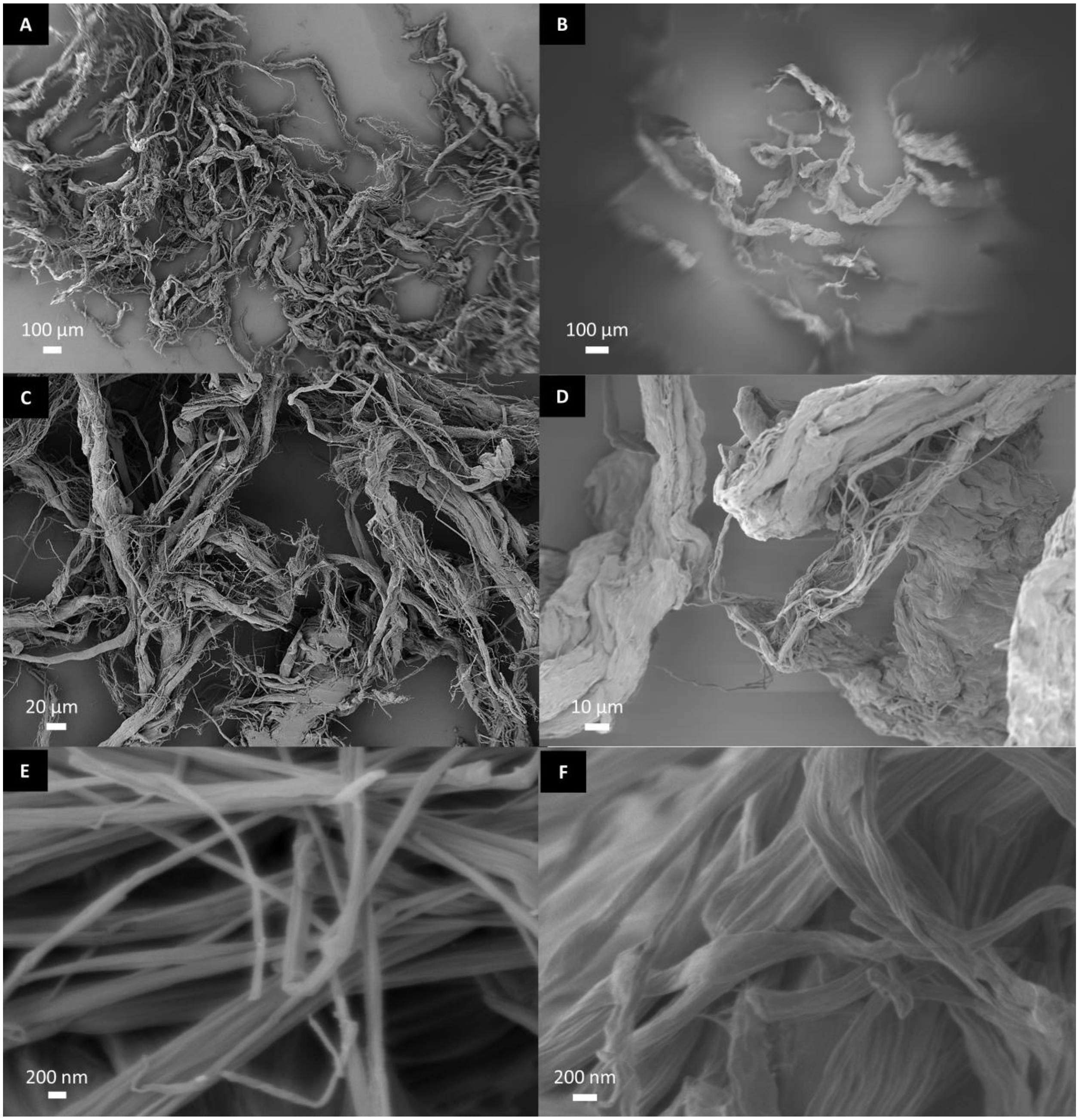

Figure S6. SEM micrographs of butanol product after vacuum filtration. (A), (C), and (E) were formed by mixing 1DL colloid and butanol by hand shaking. (B), (D), and (F) were formed by mixing 1DL colloid and butanol by vortex mixer. Note the similarities between the products formed by the different processing methods.

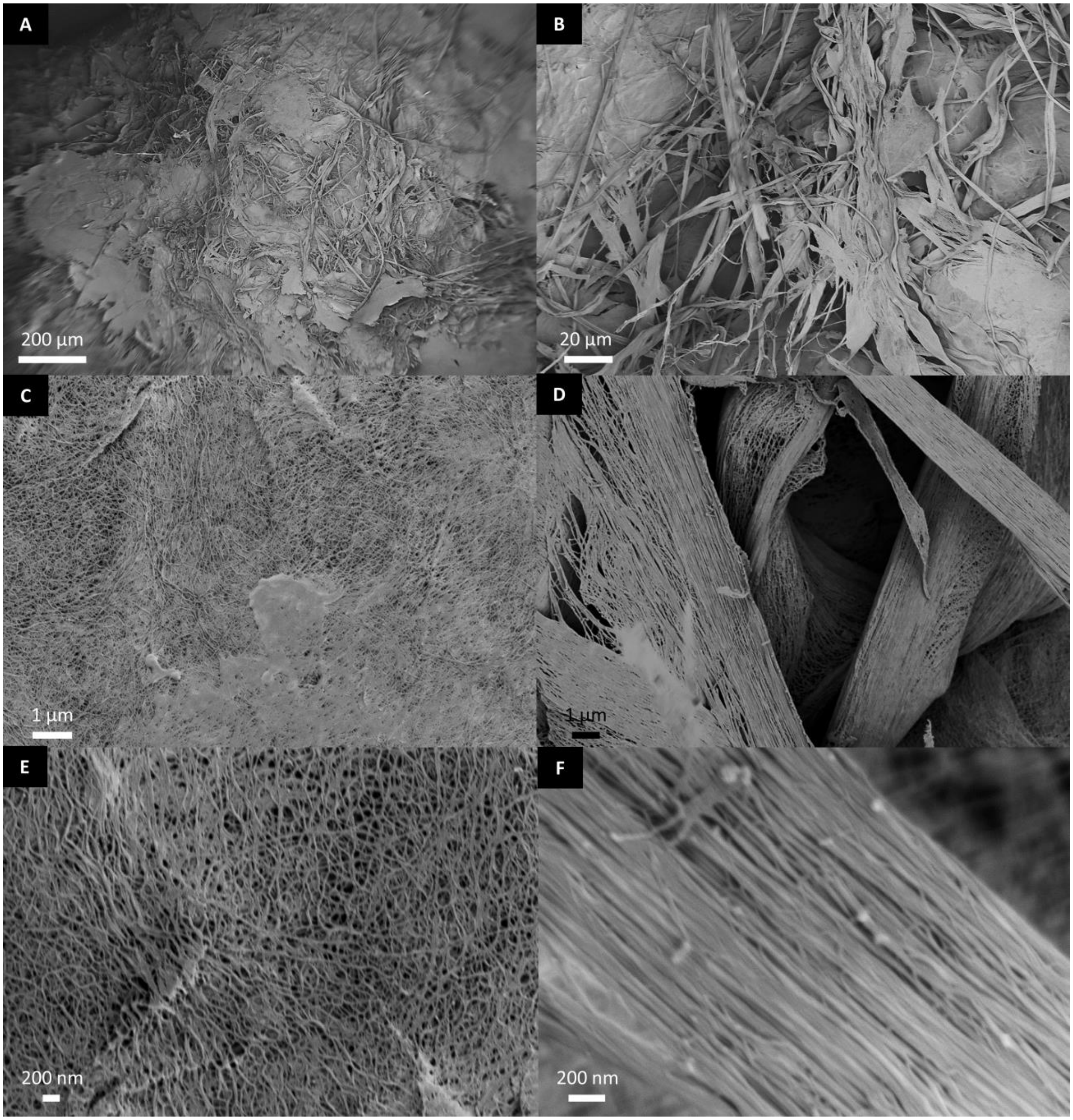

Figure S7. SEM micrographs of tert-butanol product after vacuum filtration.

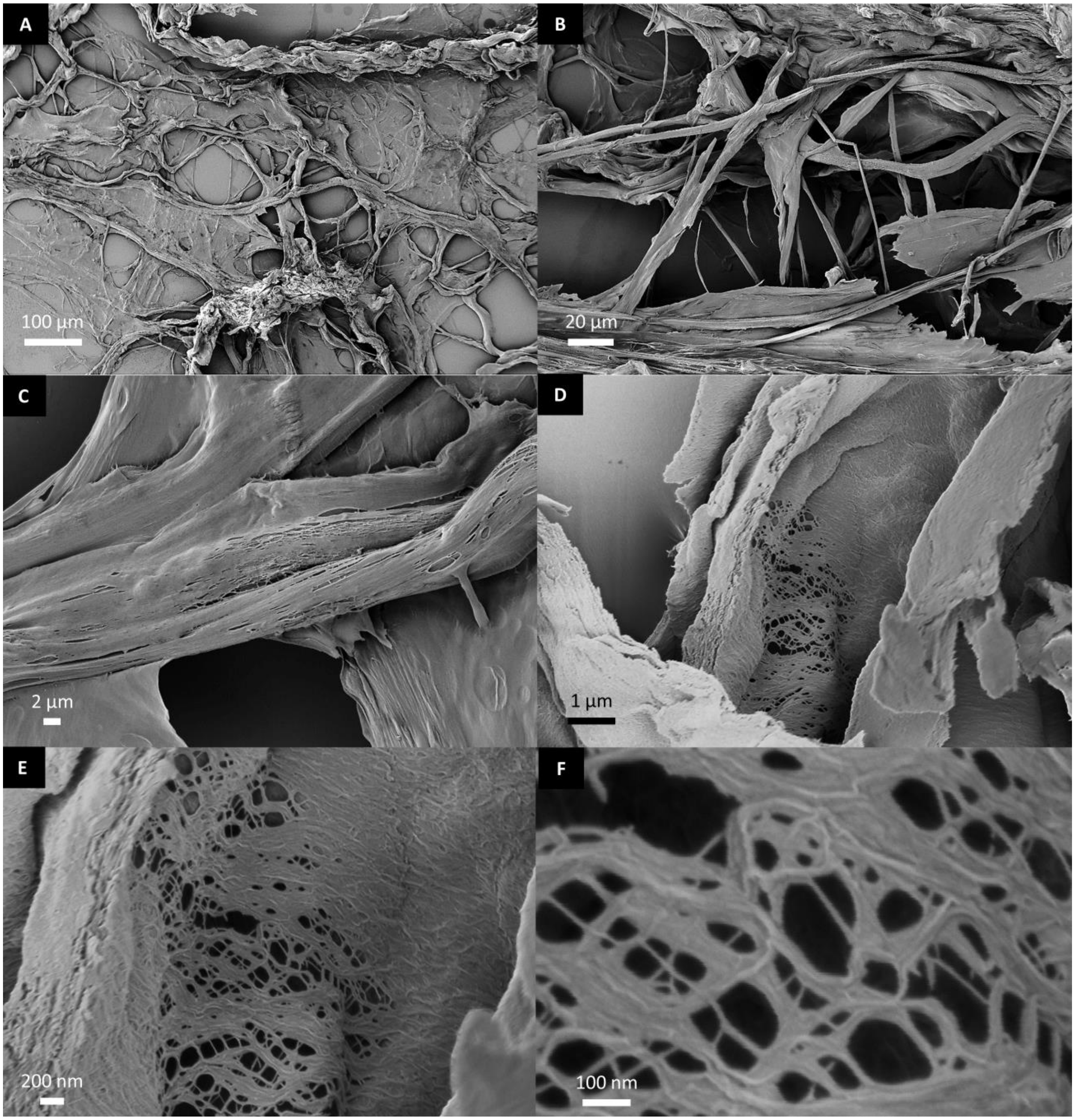

Figure S8. SEM micrographs of acetone product after vacuum filtration.

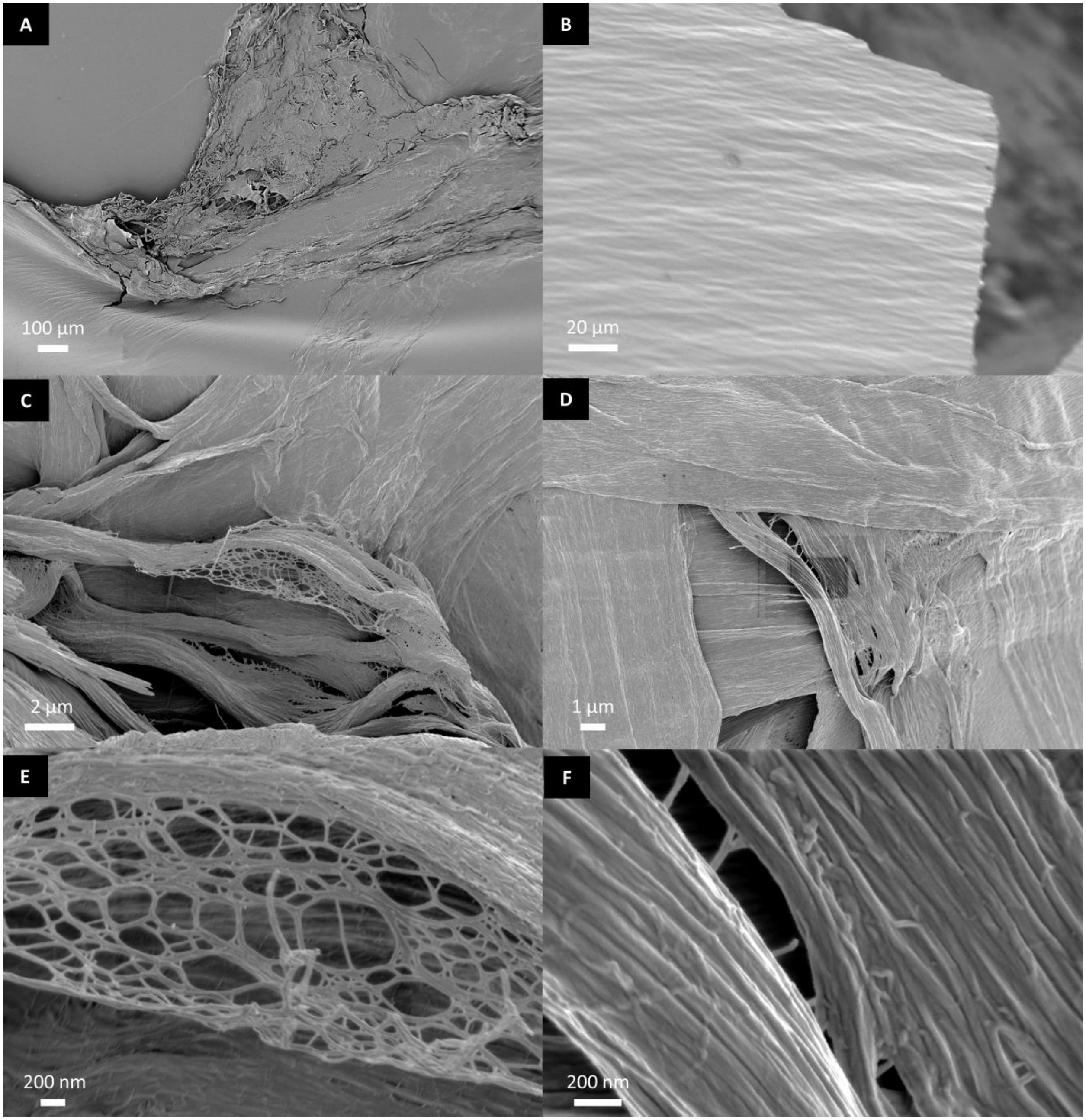

Figure S9. SEM micrographs of acetonitrile product after vacuum filtration.

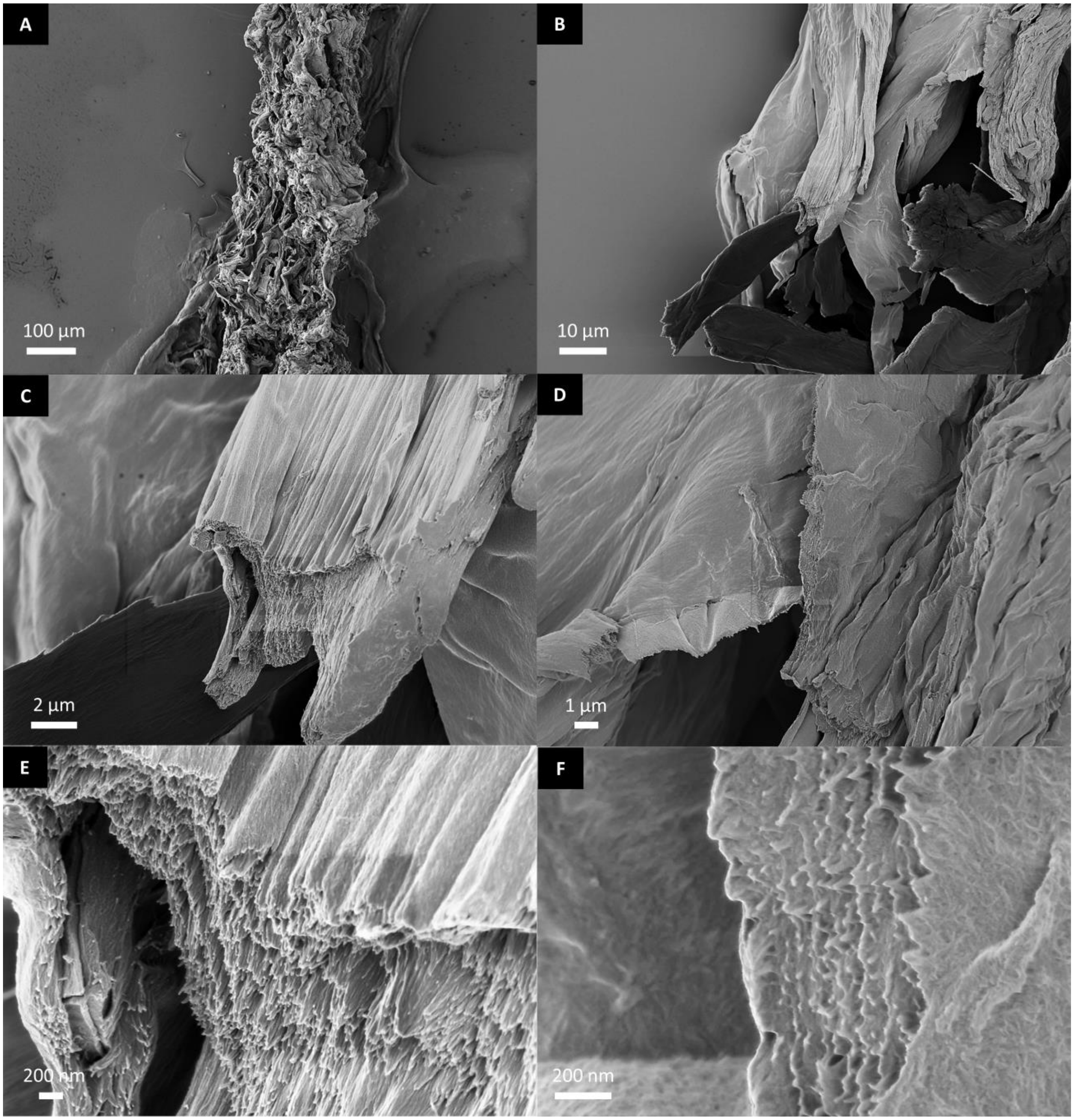

Figure S10. SEM micrographs of dimethylformamide (DMF) product after vacuum filtration.

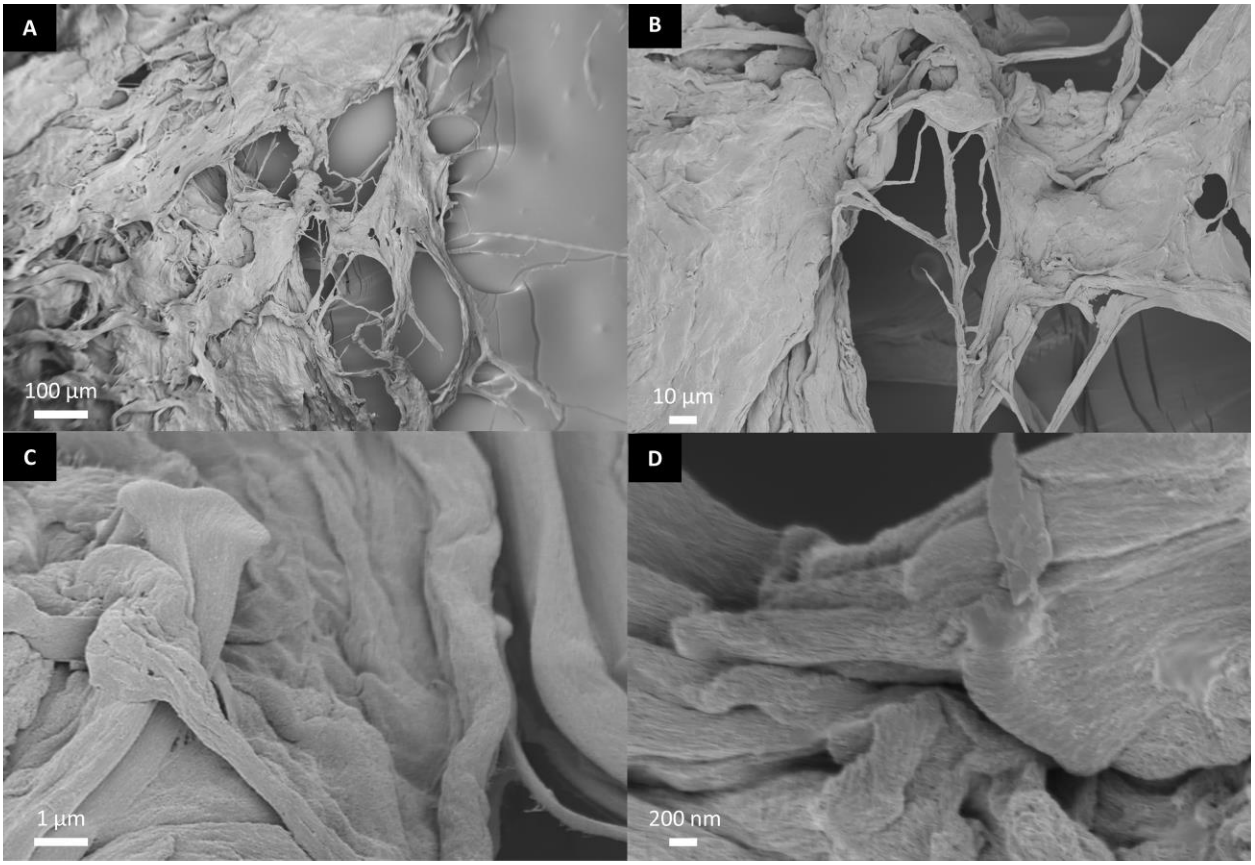

Figure S11. SEM micrographs of n-methyl-2-pyrrolidone (NMP) product after vacuum filtration.

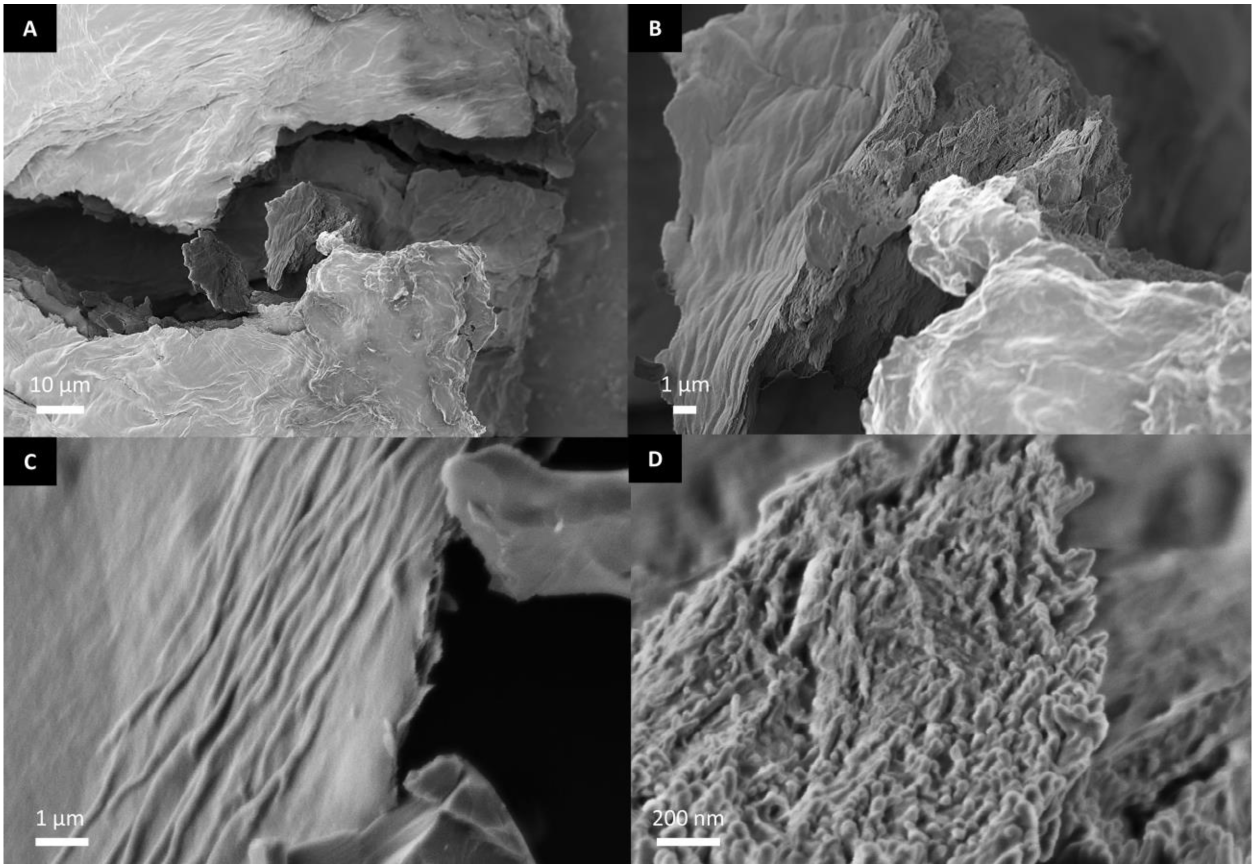

Figure S12. SEM micrographs of dimethyl sulfoxide (DMSO) product after vacuum filtration.

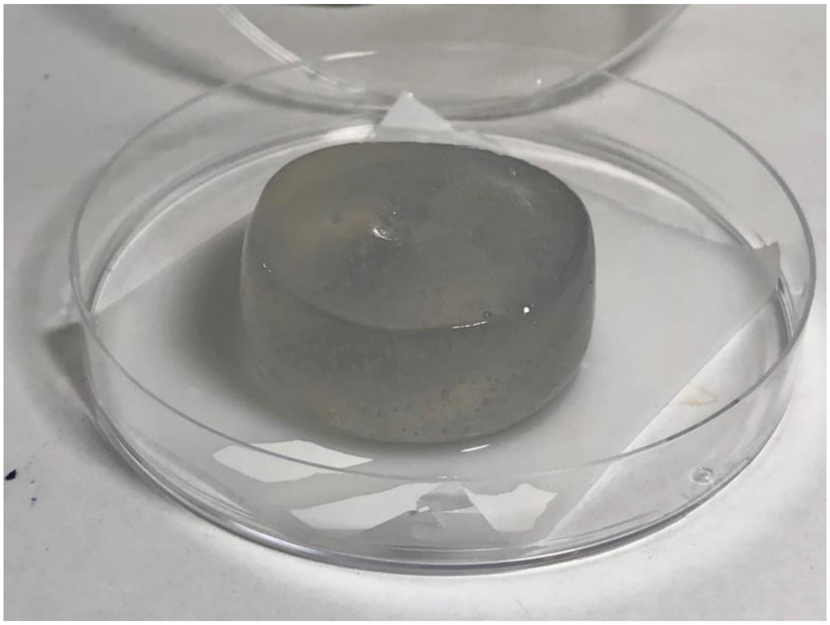

Figure S13. Gel monolith produced by combining colloidal 1DL with methanol in a 1 to 4 volume ratio, respectively. This gel was produced in a 250 mL polyethylene bottle and removed for imaging. Diameter of gel is ≈5 cm.

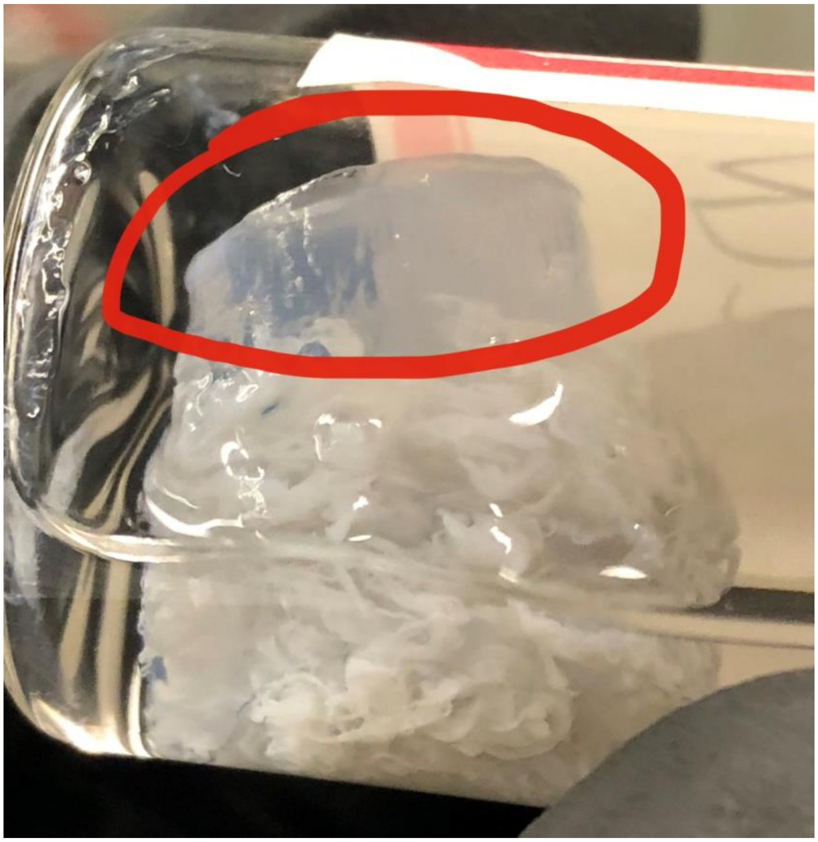

Figure S14. Gel produced by combining colloidal 1DL with acetonitrile in a 1 to 4 volume ratio, respectively. Gel was produced in a 20 mL glass scintillation vial. The red oval surrounds the soft, self-standing portion of the sample. Solid below that monolith is quite soft and resembles tentacles.

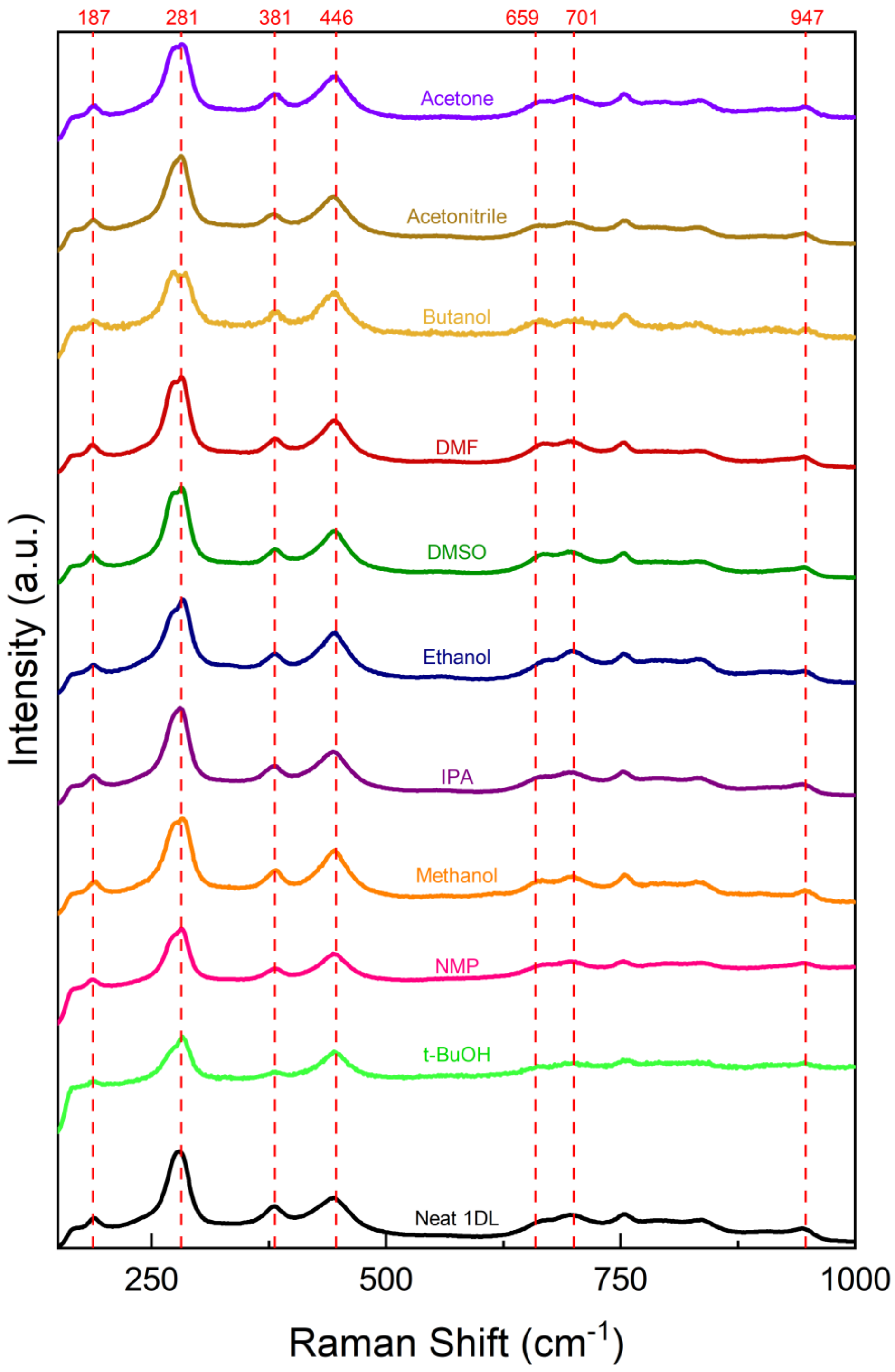

Figure S15. Raman spectra of the various films produced in this study. Note the labeled peaks are relatively unchanged across the samples. Labeled peaks are standard issue lepidocrocite[3].

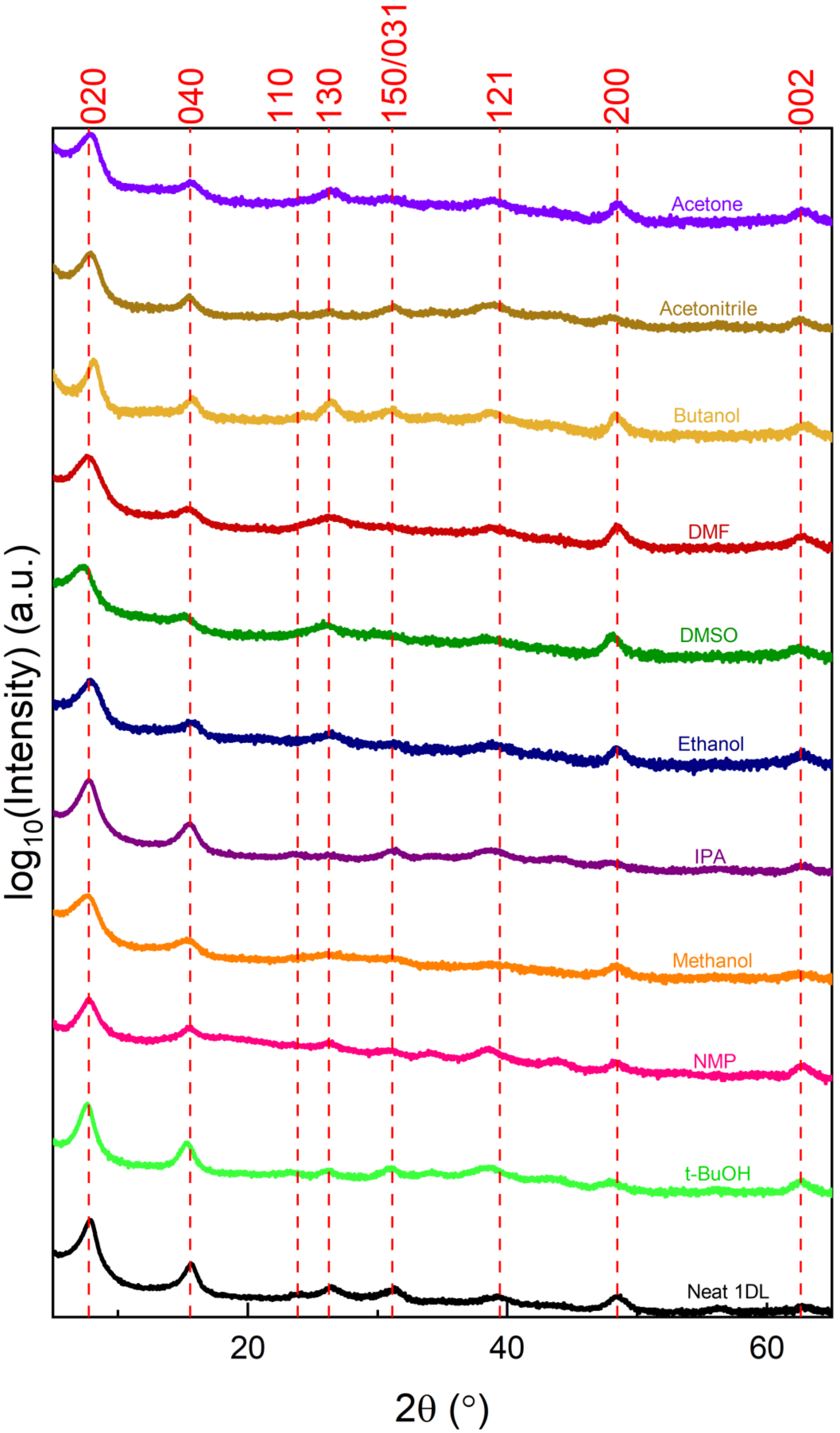

Figure S16. XRD patterns of the various films produced in this study. Note the labeled peaks are relatively unchanged across the samples. Labels at the top indicate the indexed peak values.

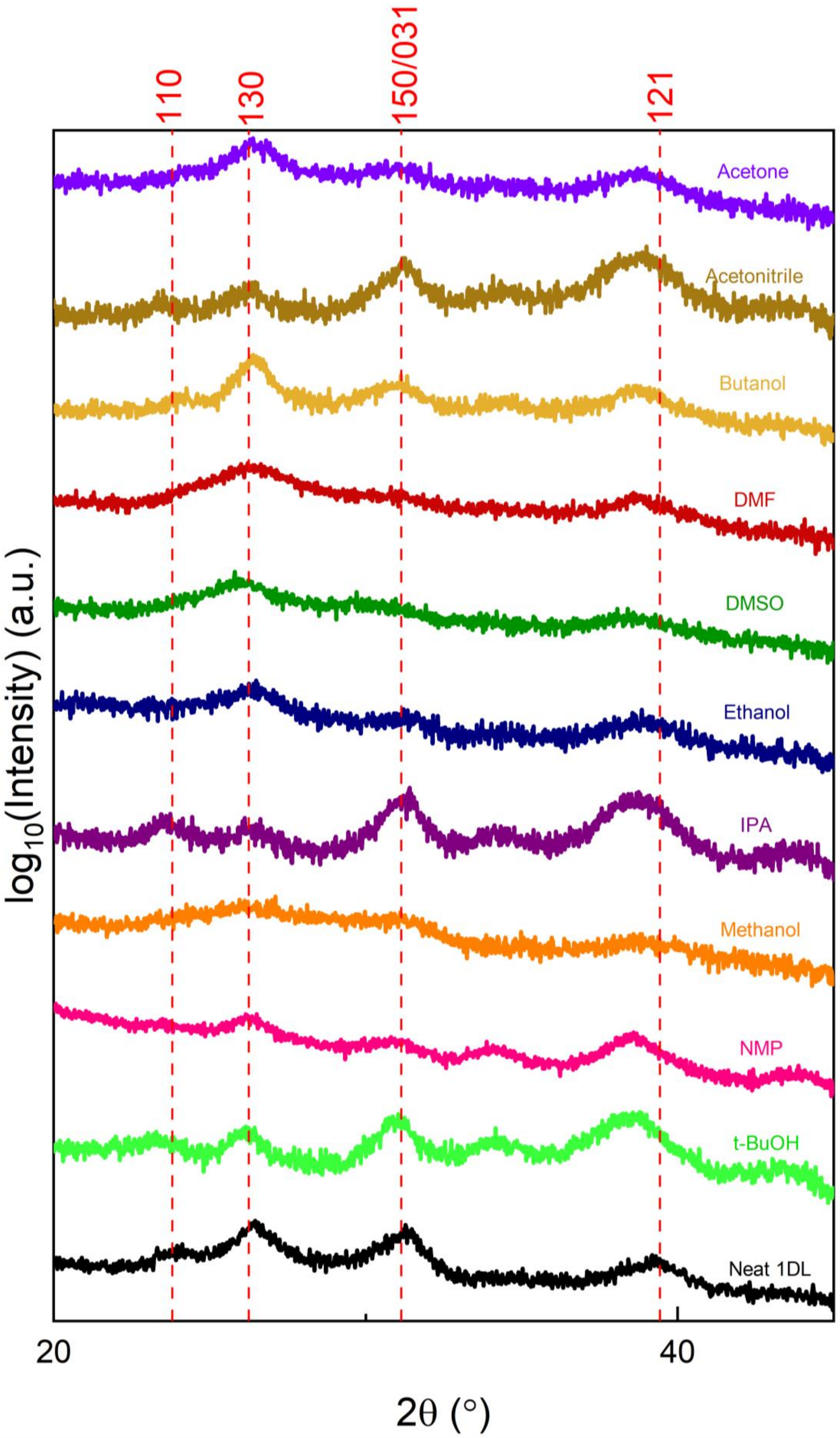

Figure S17. Zoomed in mid-region of XRD shown in **Figure S16**. Note shifting peaks in this region signifying changes in the non-stacking order peaks.

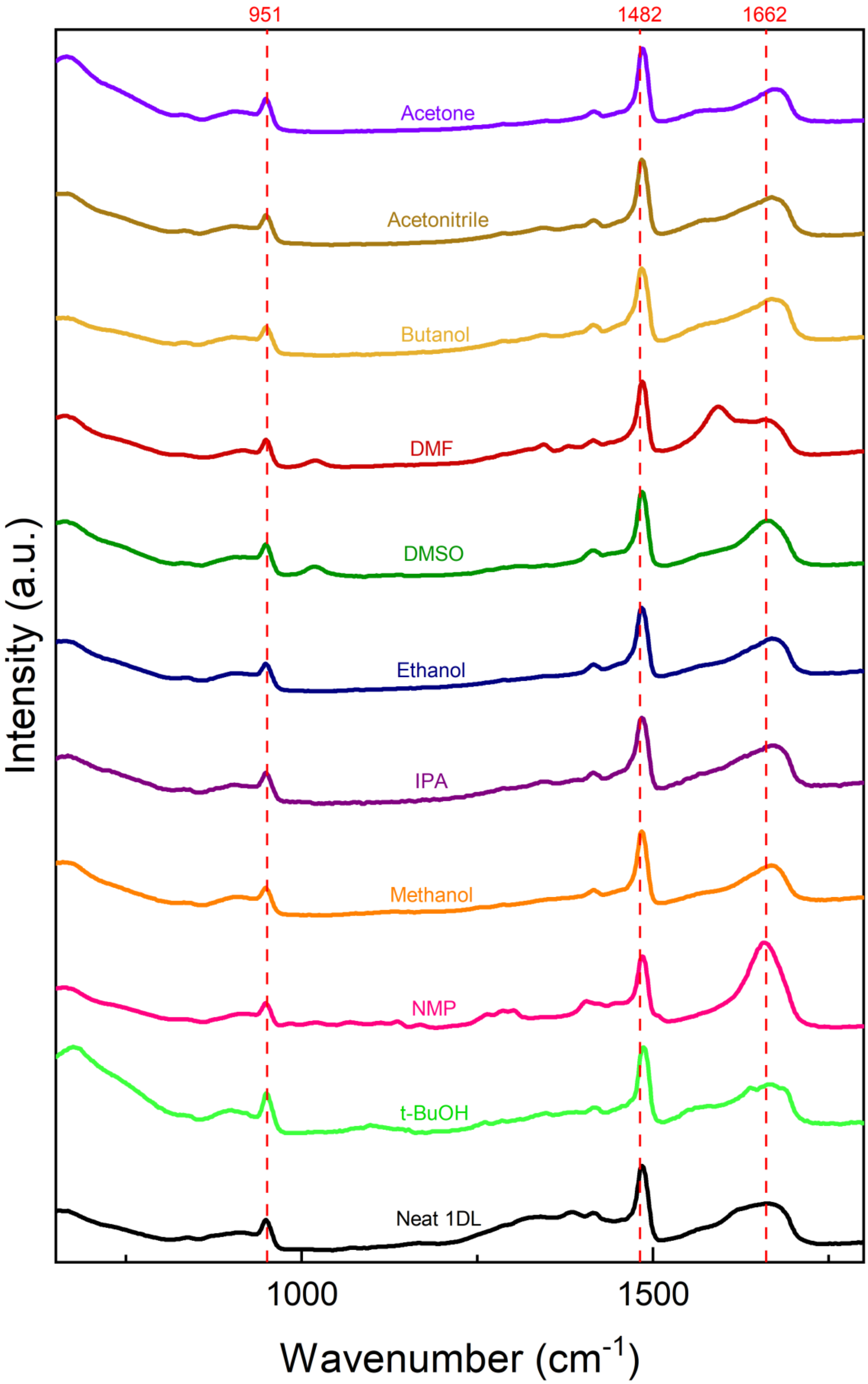

Figure S18. Low wavenumber region of the FTIR shown in **Figure 4**.

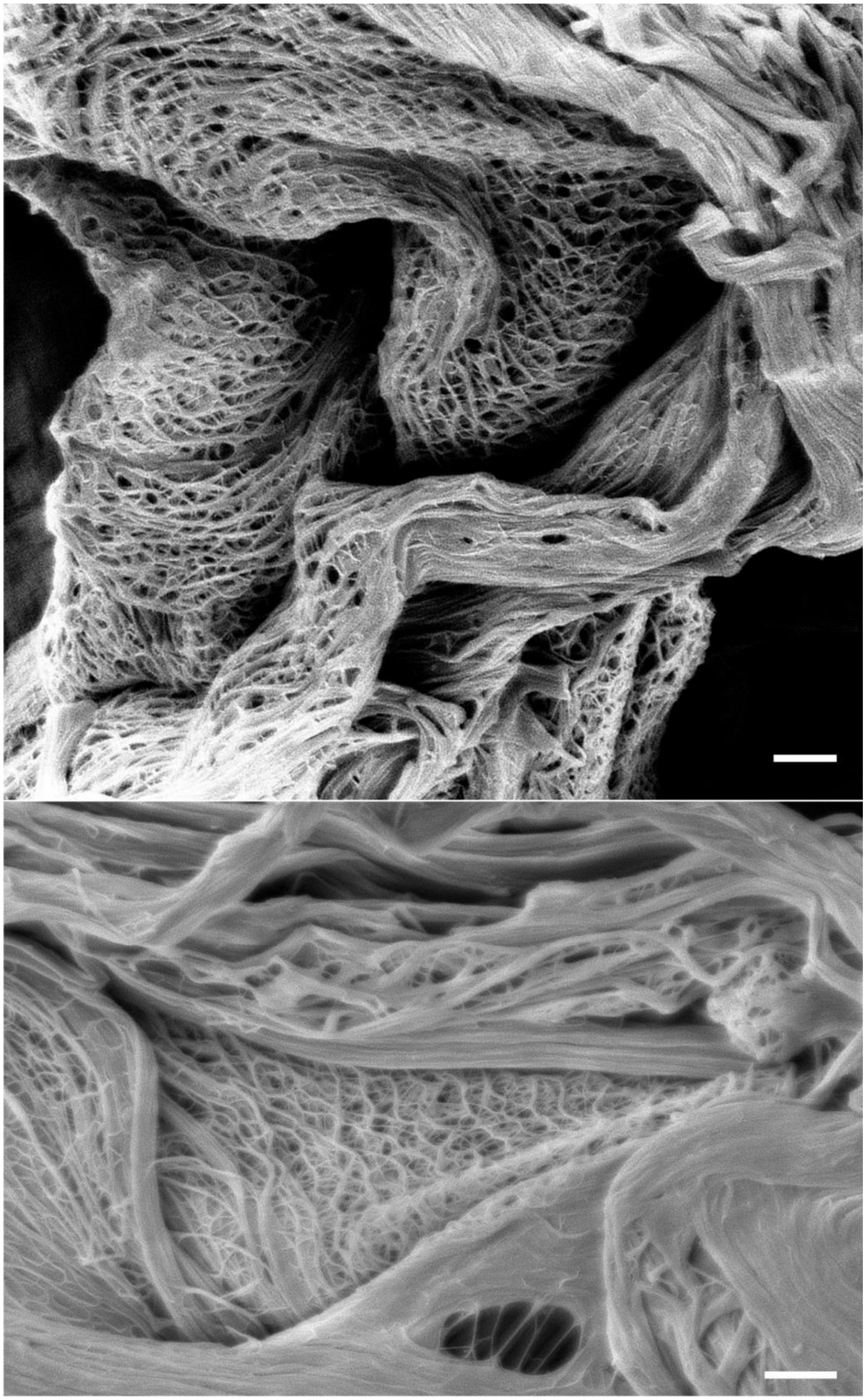

Figure S19. SEM micrographs of 1/1 w/w isopropanol/butanol product after vacuum filtration. Both scale bars are 2 μm.

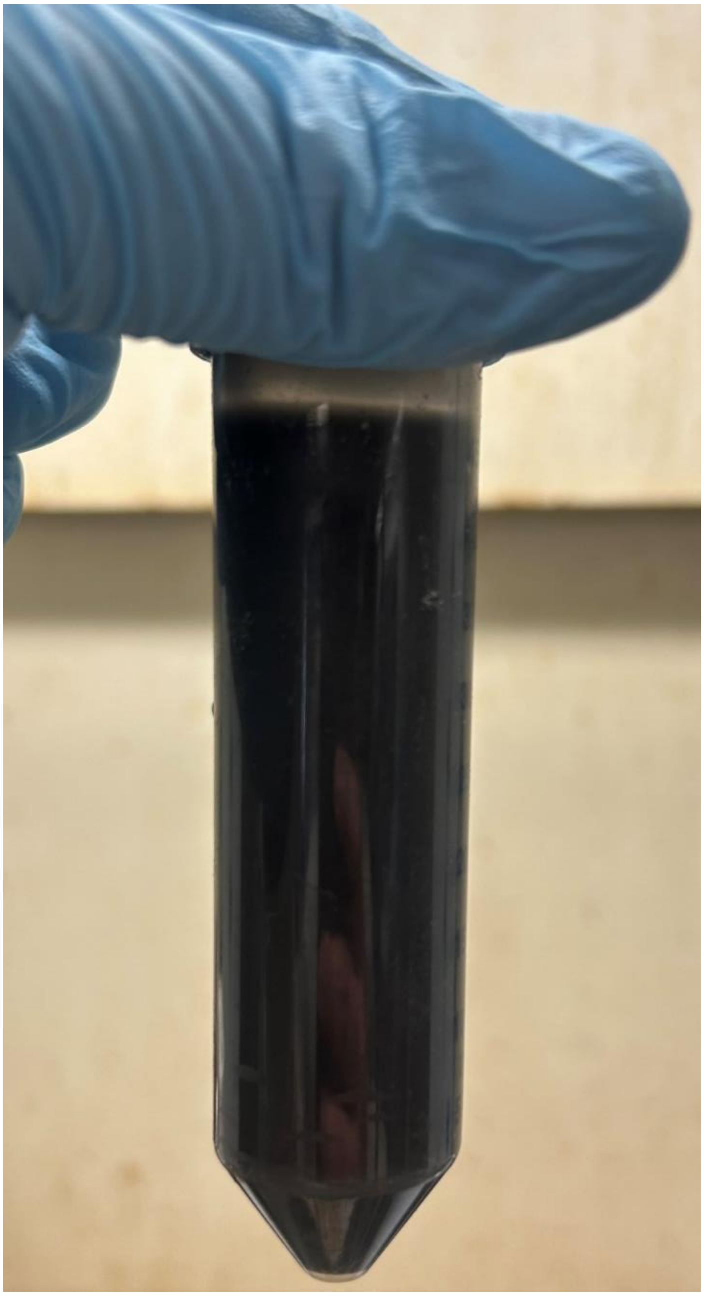

Figure S20. Photo of the concentrated (≈40 g/L) aqueous colloidal suspension of 1DLs. The suspension is a dark grey, bordering on black, with a high viscosity.

SUPPORTING REFERENCES

(1) Badr, H. O.; El-Melegy, T.; Carey, M.; Natu, V.; Hassig, M. Q.; Johnson, C.; Qian, Q.; Li, C. Y.; Kushnir, K.; Colin-Ulloa, E.; et al. Bottom-up, scalable synthesis of anatase nanofilament-based two-dimensional titanium carbo-oxide flakes. Materials today (Kidlington, England) 2022, 54, 8-17. DOI: https://doi.org/10.1016/j.mattod.2021.10.033.

(2) Badr, H. O.; Lagunas, F.; Autrey, D. E.; Cope, J.; Kono, T.; Torita, T.; Klie, R. F.; Hu, Y.-J.; Barsoum, M. W. On the structure of one-dimensional TiO2 lepidocrocite. Matter 2022. DOI: https://doi.org/10.1016/j.matt.2022.10.015.

(3) Gao, T.; Fjellvåg, H.; Norby, P. Crystal Structures of Titanate Nanotubes: A Raman Scattering Study. Inorg. Chem. 2009, 48 (4), 1423–1432. https://doi.org/10.1021/ic801508k.
